# Exploring in-person self-led debriefings for groups of learners in simulation-based education: an integrative review

**DOI:** 10.1186/s41077-023-00274-z

**Published:** 2024-01-16

**Authors:** Prashant Kumar, Susan Somerville

**Affiliations:** 1 Department of Medical Education, NHS Greater Glasgow & Clyde, Glasgow, Scotland, UK; 2 Centre for Medical Education & Dundee Institute for Healthcare Simulation, School of Medicine, University of Dundee, Dundee, Scotland, UK; 3 School of Medicine, Dentistry & Nursing, University of Glasgow, University Avenue, Glasgow, G12 8QQ Scotland, UK

**Keywords:** Debriefing, Self-led debriefing, Self-debriefing, Facilitator-led debriefing, Immersive simulation, Simulation-based education

## Abstract

**Background:**

Facilitator-led debriefings are well-established for debriefing groups of learners in immersive simulation-based education. However, there has been emerging interest in self-led debriefings whereby individuals or groups of learners conduct a debriefing themselves, without the presence of a facilitator. How and why self-led debriefings influence debriefing outcomes remains undetermined.

**Research aim:**

The aim of this study was to explore how and why in-person self-led debriefings influence debriefing outcomes for groups of learners in immersive simulation-based education.

**Methods:**

An integrative review was conducted, searching seven electronic databases (PubMed, Cochrane, Embase, ERIC, SCOPUS, CINAHL Plus, PsychINFO) for peer-reviewed empirical studies investigating in-person self-led debriefings for groups of learners. Data were extracted, synthesised, and underwent reflexive thematic analysis.

**Results:**

Eighteen empirical studies identified through the search strategy were included in this review. There was significant heterogeneity in respect to study designs, aims, contexts, debriefing formats, learner characteristics, and data collection instruments. The synthesised findings of this review suggest that, across a range of debriefing outcome measures, in-person self-led debriefings for groups of learners following immersive simulation-based education are preferable to conducting no debriefing at all. In certain cultural and professional contexts, such as postgraduate learners and those with previous debriefing experience, self-led debriefings can support effective learning and may provide equivalent educational outcomes to facilitator-led debriefings or self-led and facilitator-led combination strategies. Furthermore, there is some evidence to suggest that self-led and facilitator-led combination approaches may optimise participant learning, with this approach warranting further research. Reflexive thematic analysis of the data revealed four themes, promoting self-reflective practice, experience and background of learners, challenges of conducting self-led debriefings and facilitation and leadership. Similar to facilitator-led debriefings, promoting self-reflective practice within groups of learners is fundamental to how and why self-led debriefings influence debriefing outcomes.

**Conclusions:**

In circumstances where simulation resources for facilitator-led debriefings are limited, self-led debriefings can provide an alternative opportunity to safeguard effective learning. However, their true value within the scope of immersive simulation-based education may lie as an adjunctive method alongside facilitator-led debriefings. Further research is needed to explore how to best enable the process of reflective practice within self-led debriefings to understand how, and in which contexts, self-led debriefings are best employed and thus maximise their potential use.

**Supplementary Information:**

The online version contains supplementary material available at 10.1186/s41077-023-00274-z.

## Background

As a medium for deliberate reflective practice, debriefing is commonly cited as one of the most important aspects for learning in immersive simulation-based education (SBE) [1–3]. Defined as a ‘discussion between two or more individuals in which aspects of performance are explored and analysed’ ([4], p., 658), debriefing should occur in a psychologically safe environment for learners to reflect on actions, assimilate new information with previously constructed knowledge, and develop strategies for future improvement within their real-world context [5, 6]. Debriefings are typically led by facilitators who guide conversations to ensure content relevance and achievement of intended learning outcomes (ILOs) [7]. The quality of debriefing is thought to be highly reliant on the skills and expertise of the facilitator [8–11], with some commentators arguing the skill of the facilitator as the strongest independent predictor of successful learning [2]. Literature from non-healthcare industries tend to support this notion, suggesting that facilitators enhance reflexivity, concentration, and goal setting, whilst contributing to the creation and maintenance of psychological safety, leading to improved debriefing effectiveness [12, 13]. However, this interpretation is not universally held and has been increasingly challenged [14–18].

It is in this context that there has been an emergence of self-led debriefings (SLDs) in SBE. There is currently no consensus definition of SLDs within the literature, with the term encompassing a variety of heterogenous practices, thus causing a confusing narrative for commentators to navigate as they report on debriefing practices. We have therefore defined ‘self-led debriefing’ as debriefings conducted by the learners themselves without the immediate presence of a trained faculty member. Several reviews have investigated the overall effectiveness of debriefings, with a select few drawing comparisons between SLDs and facilitator-led debriefings (FLDs) as part of their analysis [2–4, 7, 10, 17, 19–22]. The consensus from these reviews is that there is limited evidence of superiority of one approach over the other. However, only four of these reviews conducted a critical analysis of the presence of facilitators within debriefings [2, 19, 20, 22]. Moreover, in one review [19], a narrow search strategy identified only one study comparing SLDs with FLDs [14]. To our knowledge, only one published review has explored SLDs specifically, investigating whether the presence of a facilitator in individual learner debriefings, in-person or virtual, impacted on effectiveness [23]. Within these parameters, the review concluded equivalent outcomes for well-designed SLDs and FLDs, however did not explore the influence of in-person SLDs on debriefing outcomes for groups of learners in immersive SBE. The value and place of SLDs within this context, either in isolation or in comparison with FLDs, therefore warrants further investigation.

Within the context of immersive SBE, and in-person group debriefings specifically, we challenge the concept of ‘one objective reality’, instead advocating for the existence of multiple subjective realities constructed by individuals or groups. The experiences of learners influence both their individual and group perceptions of reality and therefore different meanings may emerge from the same nominal simulated learning event (SLE) [24]. As such, this study has been undertaken through the lens of both constructionism and constructivism, with key elements deriving from both paradigms. Constructionism espouses the profound impact that societal and cultural norms have on determining how subjective experiences influence an individual’s formation of meaning within the world, or context, that they inhabit [25, 26]. Constructivism is a paradigm whereby, from their subjective experiences, individuals socially construct concepts and schemas to cultivate personal meanings and develop a deeper understanding of the world [26, 27]. In the context of in-person group debriefings, the creation of such meaning, and therefore learning, may be shaped and influenced by the presence or absence of facilitators [24].

The discourse surrounding requirements for facilitators and their level of expertise in debriefings has important implications due to the resources required to support faculty development programmes [2, 8, 9, 28]. SLDs are a relatively new concept offering a potential alternative to well-established FLD practices. Evidence exploring the role of in-person SLDs for groups of learners in immersive SBE is emerging but is yet to be appropriately synthesised. The aim of this integrative review (IR) is to collate, synthesise and analyse the relevant literature to address a gap in the evidence base, thereby informing simulation-based educators of best practices. The research question is: *with comparison to facilitator-led debriefings, how and why do in-person self-led debriefings influence debriefing outcomes for groups of learners in immersive simulation-based education?*

## Methods

The traditional perception of systematic reviews as the gold-standard review type has been increasingly challenged, especially within health professions educational research [29]. An IR systematically examines and integrates findings from studies with diverse methodologies, including quantitative, qualitative, and theoretical datasets, allowing for deep and comprehensive interrogation of complex phenomena [30]. This approach is particularly relevant in SBE, where researchers employ a plethora of study designs from differing theoretical perspectives and paradigms. An IR is therefore best suited to answer our research question and help satisfy the need for new insights such that our understanding of SBE is not restricted [31].

This IR has been conducted according to Whittemore & Knafl’s framework [30] and involved the following five steps: (1) problem identification; (2) literature search; (3) data evaluation; (4) data analysis; and (5) presentation of findings. Whilst the key elements of this study’s methods are presented here, a detailed account of the review protocol has also been published [24]. The protocol highlights the rationale and justification of the chosen methodology, explores the underpinning philosophical paradigms, and critiques elements of the framework used [24].

### Problem identification

We modified the PICOS (population, intervention/interest, comparison, outcome, study design) [32] framework to help formulate the research question for this study (Table 1), supplementing the ‘comparison’ arm with ‘context’ as described by Dhollande et al. [33]. This framework suited our study in which the research question is situated within the context of well-established FLD practices within SBE. Simultaneously, it recognises that studies with alternative or no comparative arms can also contribute valuable insights into how and why SLDs influence debriefing outcomes.

**Table 1:** PICOS framework [32, 33] used to construct research question

**Population**	In-person immersive SBE debriefing participants
**Intervention / Interest**	Self-led debriefings
**Comparison / context**	With or without facilitator-led debriefings
**Outcome**	Any outcomes
**Study design**	Integrative: both quantitative and qualitative studies included

### Literature search

#### Search strategy

Using an extensive and broad strategy to optimise both the sensitivity and precision of the search, we searched seven electronic bibliographic databases (PubMed, Cochrane, Embase, ERIC, SCOPUS, CINAHL Plus, PsychINFO), up until and including October 2022. The search terms are presented below in a logic grid (Table 2). Using a comparator/context arm minimised the risk of missing studies describing SLDs as what they are not—i.e. ‘without a facilitator’. A full delineation of each search strategy, including keywords and Boolean operators, for each electronic database is available (Additional file 1). Additionally, we conducted a manual search of reference lists from relevant studies and SBE internet resources. We enlisted the expertise of a librarian to ensure appropriate focus and rigour [34, 35].

**Table 2:** Logic grid aligned with the PICOS elements of the review question, omitting outcome/study design categories [33–35]

PICOS Framework Category	Key search terms
**Population / problem / setting**	Simulation training [MeSH], Simulation-based, Simulation-enhanced, Simulation training, Simulation teaching, Simulation event, Immersion, Simulation, Simul*, Debrief*, Conversation*
**Intervention**	Self-led, Peer-led, Group-led, Participant-led, Student-led, self-directed, Student-directed, Self-guided, Self-facilitated, Peer-facilitated, Group-facilitated, Student-facilitated, Self-debrief*, Peer-debrief*, Group-debrief*, Self debrief*, Peer debrief*, Group debrief*, Within-team
**Comparison / context**	Facilitator-led, Instructor-led, Faculty-led, Instructor debrief*, Facilitated
**Outcome**	Not applicable
**Study design**	Not applicable

#### Inclusion and exclusion criteria

Articles were included in this review if their content met the following criteria: (1) in-person debriefings following immersive simulated learning events; (2) debriefings including more than one learner; (3) healthcare professionals or students as learners; (4) published peer-reviewed empirical research; (5) reported in English. Forms of grey literature, such as doctoral theses, conference or poster abstracts, opinion or commentary pieces, letters, websites, blogs, instruction manuals and policy documents were excluded. Similarly, studies that described clinical event, individual, non-immersive or virtual debriefings were also excluded. Date of publication was not an exclusion criterion.

#### Study selection

Following removal of duplicates using bibliographical software package EndNote™ 20, we screened the titles and abstracts of retrieved studies for eligibility. Full texts of eligible studies were examined. Application of the inclusion and exclusion criteria determined which of these studies were appropriate for inclusion in this IR. We used a modified version of the Preferred Reporting Items for Systematic Review and Meta-Analysis Protocols (PRISMA) reporting tool [36] to document this process.

### Data evaluation

The process of assessing quality and risk of bias is complex in IRs due to the diversity of study designs, with each type of design generally necessitating differing criteria to demonstrate quality. In the context of this complexity, we used the Mixed Methods Appraisal Tool (MMAT) which details distinct criteria tailored across five study designs: qualitative, quantitative randomised-controlled trials (RCTs), quantitative non-RCTs, quantitative descriptive and mixed methods [37].

### Data analysis

Data was analysed using a four-phase constant comparison method originally described for qualitative data analysis [38, 39]. Data are compared item by item so that similar data can be categorised and grouped together, before further comparison between different groups allows for an analytical synthesis of the varied data originating from diverse methodologies. These phases include (1) data reduction; (2) data display; (3) data comparison; and (4) conclusion drawing and verification [30, 38, 39]. Following data reduction and extraction, we performed reflexive thematic analysis (RTA) according to Braun & Clarke’s [40] framework to identify patterns, themes and relationships that could help answer our research question and form new perspectives and understandings of this complex topic [41]. RTA is an approach underpinned by qualitative paradigms, in which researchers have a central and active role in the interpretative analysis of patterns of data and their meanings, and thus subsequent knowledge formation [40]. RTA is particularly suited to IRs exploring how and why complex phenomena might exist and relate to one another, as it enables researchers to analyse diverse datasets reflexively. It can therefore facilitate the construction of unique insights and perspectives that may otherwise be missed through other forms of data analysis. A comprehensive justification, explanation and critique of this process can be found in the accompanying IR protocol [24].

## Results

### Study selection and quality assessment

**Fig. 1:**
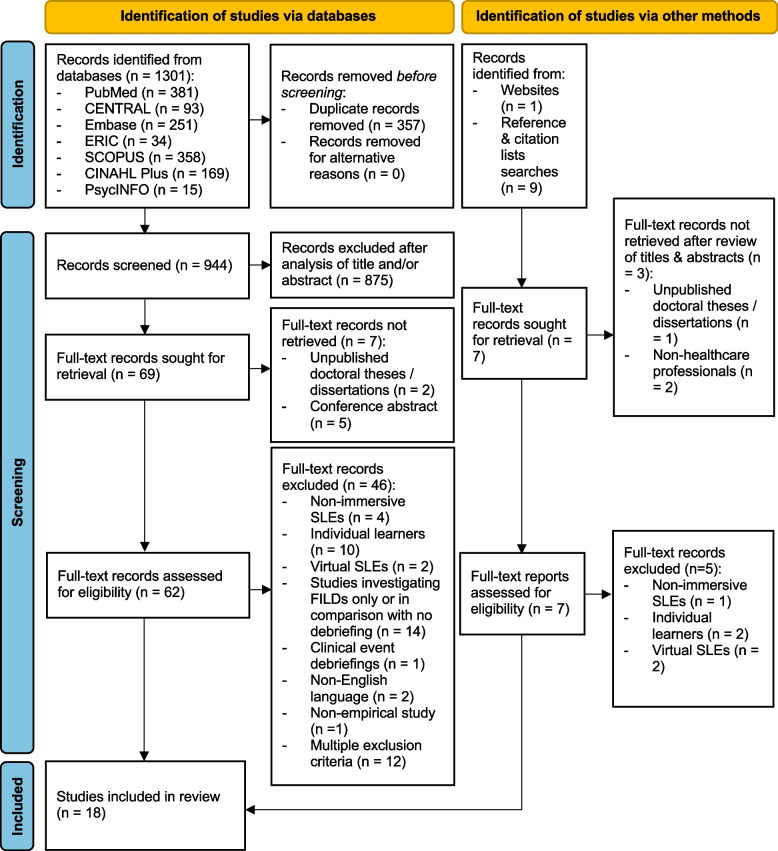
Modified PRISMA flow diagram detailing summary report of search strategy [36]

The search revealed a total of 1301 publications, of which 357 were duplicates. After screening titles and abstracts, 69 studies were identified for full-text screening. From this, a total of 18 studies were included for data extraction and synthesis (Fig. 1). Reasons for study exclusion are listed in Additional file 2.

All 18 studies were appraised using the MMAT (Table 3). Five questions, adjusted for differing study designs, were asked of each study, and assessed as ‘yes’, ‘no’ or ‘can’t tell’. The methodological qualities and risk of bias within individual studies impacted the analysis of their data and the subsequent weighting and contribution to the results of this review. The quality assessment process therefore influences the interpretations that can be drawn from such a collective dataset. Whilst the studies demonstrated varying quality, scoring between 40 and 100% of ‘yes’ answers across the five questions, no studies were excluded from the review based on the quality assessment. There were wide discrepancies in the quality of different components of the mixed methods studies. For example, Boet et al. [15] scored 0% for the qualitative component and 100% for the quantitative component of their mixed methods study. The quantitative results were therefore weighted more significantly than the qualitative component in the data analysis and its incorporation into the results of this review. Meanwhile, Boet et al.’s [16] qualitative study scored 100%, thus strengthening the influence and contribution of the results from that study within this IR.

**Table 3:** MMAT data evaluation of included empirical studies [37]

Study design	Question 1	Question 2	Question 3	Question 4	Question 5
**Qualitative studies**	**Is the qualitative approach appropriate to answer the research question?**	**Are the qualitative data collection methods adequate to address the research question?**	**Are the findings adequately derived from the data?**	**Is the interpretation of results sufficiently substantiated by data?**	**Is there coherence between qualitative data sources, collection, analysis, and interpretation?**
Boet et al. (2013) [15] – qualitative component	No	No	Can’t tell	No	No
Boet et al. (2016) [16]	Yes	Yes	Yes	Yes	Yes
Quick (2016) [42] – qualitative component	Yes	No	Can’t tell	Yes	Yes
**Quantitative RCTs**	**Is randomization appropriately performed?**	**Are the groups comparable at baseline?**	**Are there complete outcome data?**	**Are outcome assessors blinded to the intervention provided?**	**Did the participants adhere to the assigned intervention?**
Andrews et al. (2019) [43]	Can’t tell	Can’t tell	Yes	No	Yes
Boet et al. (2013) [15] – RCT component	Yes	Yes	Yes	Yes	Yes
Ha (2020) [44]	No	Yes	Yes	No	Yes
Ha & Lim (2018) [45]	Can’t tell	Can’t tell	Yes	No	Yes
Kim & De Gange (2018) [46]	Can’t tell	Yes	Yes	No	Yes
Kündig et al. (2020) [47]	Can’t tell	Can’t tell	Yes	Can’t tell	Yes
Oikawa et al. (2016) [48]	No	No	Yes	Yes	Yes
Rueda-Medina et al. (2020) [49]	No	Yes	Yes	No	Yes
Rueda-Medina et al. (2021) [50]	No	Yes	Yes	No	Yes
**Quantitative non-RCTs**	**Are the representatives of the target population appropriate?**	**Are measurements appropriate regarding both the outcome and intervention (or exposure)?**	**Are there complete outcome data?**	**Are the confounders accounted for in the design and analysis?**	**During the study period, is the intervention administered (or exposure occurred) as intended?**
Kang & Yu (2018) [51]	Yes	Yes	Yes	Can’t tell	Yes
Lee et al. (2020) [52]	Yes	Yes	Yes	Can’t tell	Yes
Na & Roh (2021) [53]	Yes	Yes	Yes	Can’t tell	Yes
Paige et al. (2021) [54]	Yes	Yes	Yes	Can’t tell	Yes
Schreiber et al. (2020) [55]	Yes	No	Yes	Can’t tell	Yes
Tutticci et al. (2017) [56]	Yes	Yes	No	Can’t tell	Yes
**Quantitative descriptive studies**	**Is the sampling strategy relevant to address the research question?**	**Is the sample representative of the target population?**	**Are the measurements appropriate?**	**Is the risk of nonresponse bias low?**	**Is the statistical analysis appropriate to answer the research question?**
Curtis et al. (2016) [57]	Yes	Yes	No	Can’t tell	No
Quick (2016) [42]– quantitative component	Yes	Yes	Yes	Yes	Yes
**Mixed methods studies**	**Is there an adequate rationale for using a mixed methods design to address the research question?**	**Are the different components of the study effectively integrated to answer the research question?**	**Are the outputs of the integration of qualitative and quantitative components adequately interpreted?**	**Are divergence and inconsistencies between quantitative and qualitative results adequately addressed?**	**Do the different components of the study adhere to the quality criteria of each tradition of the methods involved?**
Boet et al. (2013) [15]	No	No	No	Yes	No (RCT – Yes // Qualitative – No)
Quick (2016) [42]	Yes	Yes	Yes	Yes	No (RCT – Yes // Qualitative – No)

### Study characteristics

Key characteristics of articles, including the study aim and design, sample characteristics, descriptions of SLE and SLD formats, data collection instruments, and key reported study findings, are summarised in Table 4. The search elicited one qualitative study, eight quantitative RCTs, six quantitative non-RCTs, one quantitative descriptive study and two mixed methods studies. All 18 studies originated from socio-economically developed countries with six studies originating from South Korea [44–46, 51–53], five from the USA [42, 43, 48, 54, 55], and the remainder from Canada [15, 16], Australia [56, 57], Spain [49, 50], and Switzerland [47]. Two studies were multi-site [51, 52]. The immersive SLE activities were of varying formats, designs, and durations. Sixteen studies described team-based scenarios [15, 16, 42, 44–52, 54–57] whilst two used individual scenarios [43, 53], with learners then debriefing in groups of more than one learner. Four studies incorporated simulated participants in the scenarios [42, 43, 45, 46]. All studies obtained ethical approval and were published after 2013.

**Table 4:** Overview and characteristics of included studies

Authors, year, and location	Stated study aim and research design	Participant and sample characteristics	Description of SLE and SLD activity	Data collection instruments and outcome measures	Key reported study findings
	**Qualitative studies**				
Boet et al. (2016) [Toronto, Canada] [16]	To provide a narrative analysis of the content of debriefing discussion and determine the topics that facilitate reflection amongst interprofessional learners in SLDs and FLDs following a SLE. Exploratory case-study approach.	Interprofessional teams comprising of 1 anaesthesia resident (*n*=36), 1 surgical trainee (*n*=36) and 1 theatre nurse (*n*=36) (total: *n*=108) SLD: 17 teams FLD: 19 teams	Following 10-min CRM team scenario, learners commenced either 20-min SLD or FLD. Both groups then undertook a second 10-min CRM scenario, followed by FLDs. Video playback available in all debriefings. SLDs used a form based on the OGRS, with learners being asked to ‘reflect on their CRM performance and on how it could be improved’.	All SLDs and FLDs were audio-recorded and transcribed. Data were analysed qualitatively using a constant comparison method and interpreted using a social constructivist framework. Authors used a consensus-based, iterative, inductive process to identify emergent themes.	(1) 3 emergent themes revealed topics that allowed learners to enter reflection were (1) the debriefing itself, (2) experience of the simulation model (including fidelity), and (3) performance, including assessment of CRM performance. (2) SLD learners relied heavily on the OGRS form to guide their debriefing. (3) FLDs followed a more precise structure and had directed conversation, whilst SLDs had more talking over and were less structured than FLDs.
	**Quantitative RCTs**				
Andrews et al. (2019) [California, USA] [43]	To explore students’ perceived value of reviewing video recordings of, and on receiving faculty or peer feedback on, their NTS in an SLE. 2-arm prospective RCT.	3rd and 4th-year dental students (*n*=126) Sample 1 (*n*=66): SLD: *n*=45 FLD: *n*=21 Sample 2 (*n*=60): SLD: *n*=43 FLD: *n*=17	Following 20-min individual scenario with SP, learners received 5-min feedback from SP, followed by 15-min group FLD. Learners then brought back between 11 and 78 weeks later, and randomly allocated to undertake either 1-on-1 FLD (up to 40 min) or SLD with 1 other learner (up to 90 min). Both groups watched video recordings of the scenario. Sample 2 SLD learners were trained to provide constructive feedback via a 60-min session (all other groups untrained). 9 reflection questions were given to learners in both groups to guide debrief.	(1) Posttest learners questionnaires^a^ (7 items via 4-point Likert scale) (2 additional items included for SLD group). (2) Observer ratings of learner performance [ATOSCE] (sample 2 only).	(1) Learners rated FLDs significantly higher (*p*<0.05) than SLDs on most dimensions. However, both SLDs and FLDs were rated highly, with learners finding value regardless of the method. (2) Perceived value did not differ by age, gender, class year or OSCE performance. (3) Providing training for peer-feedback did not result in more favourable ratings for SLDs vs. FLDs
Ha (2020) [Chungcheongbuk-do, South Korea] [44]	To determine the effects of written versus observed SLDs when using simulation with CBL and compare levels of satisfaction between the two groups. 2-arm non-equivalent control group pretest-posttest design.	3rd-year nursing students (*n*=69) Written SLD: *n*=33 Observed SLD: *n*=36	Following 10-min team scenario, learners commenced either 20-min written SLD (using DD structure) or observed SLD (10 min watching another group undertake a scenario, prior to 10-min SLD to compare their scenario to that of the other group). Post SLDs, learners repeated the scenario, before completing questionnaires prior to undertaking a FLD.	(1) Observer ratings of learner performance pre- and posttest checklist^a^ (15 items across 6 domains). (2) Posttest learner satisfaction with SBL questionnaire^a^ (20 items across 8 domains via 5-point Likert scale). (3) Posttest learner Satisfaction with debriefing questionnaire^a^ (10 items via 5-point Likert scale).	(1) Clinical performance competency scores in both groups were significantly higher posttest vs. pretest. (2) Communication was significantly higher in the observed SLD group vs. written SLD group (*p*=0.047). There were no significant differences in any other between the 2 groups. (3) No significant differences in satisfaction with SBL (*p*=0.485) or debriefing (*p*=0.309) between the 2 groups.
Ha & Lim (2018) [Chungcheongbuk-do, South Korea] [45]	To evaluate nursing students’ knowledge and confidence in preoperative nursing skills and satisfaction with debriefing and multimode simulation using SLDs compared with FLDs. *Reported as a 2-arm quasi-experimental. However, authors report using random allocation for learners. It has therefore been included in this IR as a quantitative RCT.	3rd-year nursing students (*n*=124) SLD: *n*=62 FLD: *n*=62	Following 20-min team scenario with a manikin and SP, learners commenced either 20-min written SLD (using checklist to structure debrief) or oral FLD (not described in detail). Post debriefings, learners completed questionnaires and a repeat knowledge test 2 weeks later (at which point SLD groups were offered an oral FLD).	(1) Pre- and posttest learner written knowledge assessment^a^. (2) Posttest learner self-confidence questionnaire^a^ (4 domains via 5-point Likert scale). (3) Posttest learner satisfaction with multimode simulation questionnaire (22 items via 5-point Likert scale) (Ryoo et al., 2013). (4) Posttest learner satisfaction with debriefing [DES].	(1) No significant difference in knowledge scores pre- to posttest in both groups (*p*=0.940). (2) The self-confidence of preoperative nursing skills was statistically higher in the oral FLD group vs. written SLD group (18.81 vs. 17.85, *p*=0.010). (3) No significant difference in learner satisfaction scores with multimode simulation (*p*=0.200) and debriefing (*p*=0.423) between the 2 groups.
Kim & De Gange (2018) [Seoul, South Korea] [46]	To explore the effectiveness of SLDs and FLDs on nursing students’ knowledge, skills, self-confidence, and quality of debriefing following a preoperative care SLE using an SP. 2-arm non-equivalent control group pretest-posttest design.	3rd-year nursing students enrolled in operating room care course (*n*=57) SLD: *n*=31 FLD: *n*=26	Following 20-min team scenario with SP, learners commenced either SLD (using a written GAS framework) or FLD. Post debriefings, learners repeated the scenario, before completing questionnaires. SLD learners could then ask instructors questions or for performance feedback.	(1) Pre- and posttest learner written knowledge assessment^a^. (2) Observer ratings of learner performance pre- and posttest checklist^a^ (18 items). (3) Pre- and posttest learner self-confidence questionnaire^a^ (10 items via 10-point Likert scale). (4) Posttest learner assessment of debriefing quality [DASH-SV].	(1) There were no significant differences in knowledge of preoperative care or self-confidence from pretest to posttest in either of the groups. (2) Nursing skills for preoperative care (*p*< 0.001) was statistically higher in FLDs vs. SLDs. (3) Quality of debriefing (*p*< 0.001) was statistically higher in FLDs vs. SLDs. (3) There were no statistically significant differences in self-confidence (*p*=0.686) or knowledge (*p*=0.445) between the 2 groups.
Kündig et al. (2020) [Bern & Basel, Switzerland] [47]	To test the effects of SLDs on resuscitation performance: hands-on time, coordination between chest compressions and ventilation and defibrillation. 2-arm RCT.	4th-year medical students (*n*=171) SLD: *n*=81 No debriefing: *n*=87	Following 3-min CA team scenario, learners commenced either 3-min SLD (using written instructions on how to reflect) or 3-min no debriefing (this group performed X-ray interpretation tasks). After 3 minutes, learners commenced a second CA scenario.	(1) Observer assessment of learner performance of percentage hands-on time, coordination between chest compressions and ventilation, and defibrillation performance.	(1) Compared to the no debriefing group, learners in the SLD group showed higher performance gain in the second resuscitation scenario in percentage of hands-on time (6.21 percentage point increase, *p*< 0.001) and coordination between chest compressions and ventilation (15.0 percentage points, *p*< 0.001). However, the SLD group demonstrated a non-significantly reduced performance for defibrillation (minus 9 percentage points, *p*=0.312).
Oikawa et al. (2016) [Hawai’i, USA] [48]	To determine if learner-assessed SPA and TPA scores were different when SBE occurred with SLDs or FLDs. 2-arm prospective, controlled, cohort intervention study.	Postgraduate year 1 doctors of varying specialities (*n*=57) SLD: *n*=30 FLD: *n*=27	Following 5-min team scenario, learners commenced either 15-min SLDs (checklist-guided) or FLDs. Post debriefings, learners repeated the cycle 3 times. Following on from all 4 scenarios and debriefings, all learners attended an instructor-led course conclusion. The SLD group were instructed to independently complete a scenario-specific checklist prior to engaging in group discussion.	(1) Pre- and posttest learner assessment [TPA^b^]. (2) Pre- and posttest learner assessment [SPA^b^].	(1) Posttest scores for both TPA (*p*=0.13) and SPA (*p*=0.14) improved significantly compared to pretest scores in both groups. (2) TPA scores were significantly higher for the SLD group (14.5) than the FLD group (13.3) (*p*=0.001) whereas there was no significant difference in SPA scores (*p*=0.05).
Rueda-Medina et al. (2020) [Granada, Spain] [49]	To investigate the effectiveness of SLDs compared with FLDs or combined SLDs + FLDs, in terms of debriefing assessment, problem-solving process, and team effectiveness. 3-arm posttest-only experimental design.	Interprofessional teams of 2nd-year nursing (*n*=177), physiotherapy (*n*=39), and OT (*n*=36) students (total: *n*=252) SLD: *n*=77 SLD + FLD: *n*=90 FLD: *n*=85	Following 15-min team scenario, learners commenced either 75-min SLD, combined SLD + FLD or FLD. In the SLD and combined SLD + FLD groups, learners independently completed a questionnaire (based on GAS framework), prior to commencing group discussions (either self-led or facilitator-led), using the questionnaire as a discussion guide. Whilst video playback of the scenario occurred in the SLD, it was unclear whether it was used in the combined SLD + FLD group. The GAS framework and video playback used in the FLDs. Post debriefings, learners completed questionnaires, with SLD group then attending a FLD.	(1) Posttest learner experience with debriefing [DES]. Facilitator aspects of the scale were modified to state ‘facilitated questions’ for SLD group. (2) Posttest learner self-assessment of problem-solving abilities [PSI]. (3) Posttest learner assessment of team effectiveness [CATS].	(1) Nursing: the SLD + FLD group had a significantly higher CATS score (15.63) than either the SLD (13.91) or FLD group (13.71) (*p*< 0.001). There were no significant differences for DES or PSI between the groups. (2) Physiotherapy: the SLD + FLD group had a significantly higher CATS score (13.50) than either the SLD only group (10.83) or FLD only group (10.36) (*p*< 0.009). There were no significant differences for DES or PSI between the groups. (3) OT: there were no significant differences for DES, PSI or CATS between the groups.
Rueda-Medina et al. (2021) [Granada, Spain] [50]	To compare the debriefing assessment and satisfaction perceived by nursing students who experience SLDs, FLDs or combined SLDs + FLDs. 3-arm randomised experimental design.	Nursing students (*n*=177) SLD: *n*=58 SLD + FLD: *n*=68 FLD: *n*=51	Following a 15-min enteral-feeding team scenario, learners commenced either 75-min SLD, combined SLD + FLD or FLD. In the SLD and combined SLD + FLD groups, learners completed a questionnaire (based on GAS framework) prior to commencing group discussions (either self-led or facilitator-led), using the questionnaire as a discussion guide. GAS framework used in the FLDs. Video playback used in all groups. Post debriefings, learners completed questionnaires, with SLD group then attending a FLD.	(1) Posttest learner assessment of debriefing quality [DASH-SV]. (2) Posttest learner satisfaction with debriefing [CESS]. (3) Posttest learner satisfaction with debriefing [10-cm VAS].	(1) DASH-SV score in the SLD + FLD group was significantly higher compared with FLD group (*p*=0.03). (*2*) DASH-SV score in the FLD was significantly higher compared with the SLD group (*p*=0.08). (3) CESS score in the SLD + FLD group (146.60) was significantly higher than the SLD group (140.71) and the FLD group (136.07) (*p*=0.039). (4) VAS score in the SLD + FLD group (9.25) was significantly higher than the SLD group (8.40) and the FLD group (8.37) (*p*=0.13).
	**Quantitative non-RCTs**				
Kang & Yu (2018) [Jeju & South Gyeongsang, South Korea] [51]	To determine differences in the problem-solving process, team effectiveness, debriefing assessment, and debriefing satisfaction between SLDs + FLDs compared with FLDs only, and to determine if these are affected by number of SLD sessions. 2-arm non-equivalent control group pretest-posttest design.	4th-year nursing students (*n*=123) SLD + FLD: *n*=60 FLD: *n*=63	Following 20-min team scenario, learners commenced either 30-min SLDs (using a questionnaire based on GAS framework and video playback) or FLDs. Post debriefings, learners repeated the cycle 4 times in sequence following on from other groups. The number of SLDs performed therefore varied depending on the sequence in which the groups undertook the scenarios (some groups undertook 5 SLDs, whilst others only had 1). Following completion of all 5 scenarios and debriefings, all learners attended a large group FLD.	(1) Pre- and posttest learner self-assessment of problem-solving abilities [PSPIA]. (2) Pre- and posttest learner assessment of team effectiveness tool (via 7-point Likert scale) (Lim & Kang, 2005). (3) Posttest learner assessment of debriefing quality [DASH-SV]. (4) Posttest learner satisfaction with debriefing [10-cm VAS].	(1) The SLD + FLD group showed significant improvement in the problem-solving process (*p*<0.01) and debriefing satisfaction (*p*=0.02), but not in debriefing assessment (*p*=0.097) or team effectiveness (*p*=0.069) compared to the FLD group. (2) Groups participating in a higher number of SLDs had significant improvements in problem-solving ability (*p*<0.001) and debriefing satisfaction (*p*<0.001). Furthermore, debriefing assessment scores and team effectiveness tended to increase with the number of SLD sessions, but non-significantly.
Lee et al. (2020) [52] [Gangwon-do, South Korea]	To compare three debriefing methods (FLD, SLD, and video assisted SLD) by measuring academic self-efficacy, confidence in performance, self-assessed communication, and satisfaction. Multi-site 3-arm quasi-experimental study using a pretest-posttest design.	Senior nursing students from 3 South Korean universities (*n*=146) SLD: *n*=49 video assisted SLD: *n*=50 FLD: *n*=47	Following 20-min prematurity care team scenario, learners commenced either 90-min SLD (using a questionnaire structured via description, analysis, and application framework), video assisted SLD or FLD (using description, analysis, and application framework). In the video assisted SLD group, learners reviewed the full scenario recording (20 min), before providing written responses to the same questionnaire (15 min). This was followed by instructor feedback.	(1) Pre- and posttest learner academic self-efficacy assessment [ASES]. (2) Pre- and posttest learner self-confidence questionnaire (15 items via 5-point Likert scale) (Lee et al., 1991). (3) Pre- and posttest learner self-assessed communication skills [GICC]. (4) Posttest learner satisfaction with debriefing questionnaire (16 items via 5-point Likert scale) (Otieno et al., 2007).	(1) Academic self-efficacy (*p*=0.001), confidence in performance (*p*<0.001), and self-assessed communication skills (*p*=0.007) all improved significantly pretest to postest in all 3 groups. (2) There was no significant difference in posttest academic self-efficacy between the video assisted SLD group and the SLD and FLD groups (*p*=0.218). (3) Posttest confidence in performance was significantly higher in the video assisted SLD group vs. the SLD (−0.07) and FLD (−0.33) groups (*p*=0.001). (4) Posttest self-assessed communication skills were significantly higher in the video assisted SLD group vs. the SLD (−0.08) and FLD (−0.25) groups (*p*=0.007). (5) Posttest satisfaction with debriefing methods were significantly higher in the video assisted SLD group compared to the SLD (−0.04) and FLD (−0.51) groups (*p*< 0.001). However, satisfaction was high in all groups.
Na & Roh (2021) [Seoul, Gangwon & Chungcheong, South Korea] [53]	To compare the effects of SLDs and FLDs on cognitive load, achievement emotions, and the nursing performance of senior nursing students. 2-arm non-equivalent control group pretest-posttest design.	Senior nursing students (*n*=55) SLD: *n*=26 FLD: *n*=29	Following 10-min hyponatraemia individual scenario, groups of 5 learners commenced either 50-min SLD (led by a volunteer student using GAS framework) or FLD (using GAS framework). Both groups contained a 20-min self-reflection with journal writing element (based on GAS framework) prior to group discussion. Following debriefing, the scenario was repeated.	(1) Pre- and posttest learner self-report on cognitive load [CLMT]. (2) Pre- and posttest learner self-report on achievement emotions [AEQ-Korean version]. (3) Observer ratings of learner performance [NPC].	(1) There were no statistically significant differences between the 2 groups regarding total cognitive load (*p*=0.437), nursing performance (*p*=0.559), or the achievement emotions. (2) Nursing students in both groups showed a significantly higher overall cognitive load, higher positive and lower negative achievement emotions, and improved nursing performance after debriefing compared to before debriefing. However, when examining pre- and posttest score differences between the groups, there were no significant differences across all three measures.
Paige et al. (2021) [Louisiana, USA] [54]	To investigate the efficacy of the Q-TAS, versus the TAS, as a formative assessment of teamwork to improve the quality of SLDs. 2-arm prospective comparative analysis.	Interprofessional teams of (typically) 2 senior medical (*n*=39), 2 nursing (*n*=8), and 2 nurse anaesthesia students (*n*=23) (total: *n*=70) TAS SLD: *n*=35 Q-TAS SLD: *n*=35	Following theatre-based team scenario, learners commenced FLD. Post FLD, teams commenced a second scenario, followed by SLD. Sample 1 (2018) used the TAS tool and sample 2 (2019) used the Q-TAS tool to guide the SLDs (1 team member nominated as lead, who used the tool lead the discussion).	(1) Observer assessment of debriefing quality [OSAD].	(1) No significant difference in the overall quality of SLDs when using the Q-TAS (4.70) compared with the TAS (4.13) (*p*= 0.93). (2) A statistically significant increase in quality of the analysis segment of SLDs when using the Q-TAS (4.92) compared with the TAS (3.83) (*p*= 0.23). (3) Overall, regardless of tool used, quality of SLDs was rated highly (>4).
Schreiber et al. (2020) [Pennsylvania, USA] [55]	To examine the use of SLDs for the purposes of assessing student perception of confidence with learning and performance related feedback during SBE. 2-part modified Pretest-posttest cross-over design.	Graduate OT students (*n*=37) 1st SLE: Active participants: (*n*=18) Observers: (*n*=19) 2nd SLE (roles reversed): Active participants: (*n*=19) Observers: (*n*=18)	Following 25-min team scenario with 2 active and 2 observing learners, learners commenced 60-min SLDs (led by the observing learners using a debriefing template). This process was repeated some months later but with the learner roles switched. A facilitator was present for the first 15 min to provide guidance.	(1) Pre- and posttest learner survey assessing self-confidence and competence with learning modalities^a^ (active participation compared with observing) and with giving and receiving feedback (6 items via 7-point Likert scale).	(1) Learners perceived benefit from both active participation and observation during SBE. (2) In 1st SLE, observers believed more strongly that ‘I only learn by actively participating’ (1.05 vs. 1.17, *p*=0.025) but less strongly that ‘I am confident providing unbiased feedback to my peers’ (2.47 vs. 1.89, *p*=0.046) compared to active learners (difference not found in 2nd SLE once roles were switched). (3) In 2nd SLE, observers believed more strongly that ‘feedback I give to my peers enhances my learning’ (1.89 vs. 2.10, *p*=0.037) compared to active learners (difference not found in 1^st^ SLE). (4) Observers in 1st SLE disagreed more with the statement ‘I only learn by actively participating’ compared with observers in 2nd SLE (4.33 vs. 1.05, *p*=0.000). (5) Active learners in 1st SLE, disagreed more with the statements ‘I learn by watching others’ (2.15 vs. 1.33, *p*=0.006), ‘I only learn by actively participating’ (3.60 vs. 1.17, *p*=0.000), and ‘feedback to peers enhances my learning’ (2.10 vs. 1.33, *p*=0.003) compared with active learners in 2nd SLE.
Tutticci et al. (2017) [Queensland, Australia] [56]	To determine whether SLDs and/or SLDs + FLD differed from FLDs in assisting students to reveal critical reflection skills. 3-arm non-equivalent control group design.	Final-year nursing students (*n*=346) SLD: *n*=110 SLD + FLD: *n*=158 FLD: *n*=78	Following a chest trauma team scenario with 4 active and 4 observing learners, learners commenced either 20-min SLD (led by randomly allocated learner), SLD + FLD (co-facilitated by randomly allocated learner) or FLD. All groups required their respective facilitators to use the 5 ‘Rs’ reflective framework and checklist- reporting, responding, relating, reasoning, and reconstructing (Bain et al., 2002). All facilitators were directed to online training resources to prepare for the role of facilitator (with option to opt-out).	(1) Posttest learner assessment of reflective thinking [RTI]. (2) Posttest learner Critical Reflection Self-Efficacy [single item 0–100 VAS]. (3) Posttest learner self-confidence in coping ability [GSES]. (4) Observer assessment of debriefing quality via checklist^a^ (9 items using yes/no, including 2 items with frequency score).	(1) FLD and SLD + FLD groups had a significantly higher RTI scores vs. SLD groups (*p*=0.006). (2) There was no significant difference in the Critical Reflection Self-Efficacy VAS scores (*p*= 0.201) or the GSES scores (*p*= 0.933) between the 3 groups. (3) Debriefing checklist adherence ranged from 10.9 to 92.7% across the 9 items. No data on group adherence to data is presented.
	**Quantitative descriptive studies**				
Curtis et al. (2016) [Queensland, Australia] [57]	To evaluate peer-to-peer facilitated student led mid-level fidelity simulation experiences. Single group posttest design.	2nd and 3rd-year nursing students (*n*=509)	Following 10-min team scenario in which learners played 4 specific roles (handheld device operator, nurse, physician, and observer), learners commenced 5-min SLD (led by learner playing role of handheld operator, using 3-question approach). Following completion of all 4 scenarios and debriefings, all learners attended a large group 30-min FLD.	(1) Posttest learner satisfaction questionnaire^a^ (16 items via 6-point Likert scale). (2) Posttest learner self-confidence questionnaire (6 items adapted from SCLS).	(1) Learners self-reported high satisfaction in learning with the SLE (4.42). (2) Learners reported high self-confidence in clinical skills after exposure to the SLE (4.14).
	**Mixed methods studies**				
Boet et al. (2013) [Toronto, Canada] [15]	To test the relative effectiveness of operating room SLDs compared with FLDs for learning CRM. Two-arm RCT using repeated measures design.	Interprofessional teams of 1 anaesthesia resident (*n*=36), 1 surgical trainee (*n*=36) and 1 theatre nurse (*n*=36) (total: *n*=108) SLD: 17 teams FLD: 19 teams	Following 10-min CRM scenario, groups commenced either 20-min SLD or FLD. Both groups then undertook a second 10-min CRM scenario, followed by FLDs. Video playback available in all debriefings. SLDs used a form based on the OGRS, with learners being asked to ‘reflect on their CRM performance and on how it could be improved’.	(1) Pre- and posttest observer assessment of learners [TEAM]. (2) Transcripts of debriefings analysed using qualitatively using a constant comparison method. Detailed qualitative results published separately (Boet et al., 2016).	(1) Pre- to posttest team performance significantly improved regardless of debriefing method (*p*=0.008). (2) There was no significant difference in the degree of improvement between the groups (*p*=0.52). (3) Use of scenario video playback was similar in both groups. (4) Similar themes were discussed in both groups.
Quick (2016) [Minnesota, USA] [42]	To gain insight into the nature of the role of self- and peer-assessment in the development of dental students’ reflective practice skills with the use of an SP experience. Single group posttest analysis design.	4th year-dental students (*n*=32)	Pairs of learners undertook 4 scenarios with an SP (domestic violence, unrealistic expectations, informed consent, and breaking bad news). Learners rotated to be the active and observing learner twice each. The observer completed a worksheet during the encounter with the SP providing feedback to the active learner post-scenario. Following completion of all 4 scenarios, all learners attended a large group 35-min FLD. Learners were then re-paired such that each new pair had been the active learner with the same SP and scenario. They independently reviewed their own and their peers’ videos, before commencing a SLD (elements are not well defined beyond ‘meeting to discuss their feedback’).	(1) Posttest learner self- and peer-assessment forms, including quantitative^a^ (3 items via 1–10 scale) and qualitative (open-ended questions) data.	(1) 5 performance themes emerged: personal affect, verbal communication, professional demeanour, relationship-building, and patient management. (2) 2 student learning themes emerged: application and knowledge, ways to change, and impressed with peer/increased confidence in self. (3) Data showed that peer assessment ratings were consistently higher across the 3 items vs. self-assessments (case management: 8.09 vs. 7.25, relationship-building: 7.96 vs. 7.26, cue recognition: 8.45 vs. 7.90).

*Abbreviations: CA* cardiac arrest, *CBL* case-based learning, *CRM* crisis resource management, *DD* Diamond Debrief [58] , *FLD* facilitator-led debriefing, *GAS* Gather Analyse Summarise framework [59], *NTS* non-technical skills, *OGRS* Ottawa Global Rating Scale [60] , *OSCE* Objective Structured Clinical Examination, *OT* occupational therapy, *RCT* randomised control trial, *SBL* simulation-based learning, *SLD* self-led debriefing, *SLE* simulated learning event, *SP* simulated participant, *vs.* versus

*Data collection instruments: AEQ* Achievement Emotions Questionnaire [77 items across 8 domains, using a 5-point Likert scale] [61, 62], *ASES* Academic Self-Efficacy Scale [20 items using a 5-point Likert scale] [63], *ATOSCE* Ambulatory Team Observed Structured Clinical Evaluation [64], *CATS* Communication And Teamwork Skills [4 items using a 3-point scale] [65], *CESS* Clinical Experience Simulation Scale [17 items using a 10-point Likert scale] [66], *CLMT* Cognitive Load Measurement Tool 2.0 [16 items across 4 domains using 9- or 10-point scales] [67], *DASH-SV* Debriefing Assessment for Simulation in Healthcare- Student Version [6 items via 7-point scale] [68], *DES* Debriefing Experience Scale [20 items across 4 domains, using a 5-point Likert scale] [69], *GICC* Global Interpersonal Communication Competence scale [15 items using a 5-point Likert scale] [70], *GSES* General Self-Efficacy Scale [10 items using a 4-point Likert scale] [71], *NPC* Nursing Performance Checklist [20 items across 4 categories, using a 3-point Likert scale] [72], *OSAD* Objective Structured Assessment of Debriefing [8 items using a 5-point Likert scale] [73], *PSI* Problem Solving Inventory [35 items across 3 subscales, using a 6-point Likert scale] [74], *PSPIA* Problem Solving Process Inventory for Adults [30 items across 5 domains, using a 5-point Likert scale] [75], *Q-TAS* Quick Teamwork Assessment Scale [54], *RTI* Reflective Thinking Instrument [15 items across 4 domains using a 5-point Likert scale] [76, 77], *SCLS* Self-Confidence in Learning Scale [78], *SPA* self-assessment performance [3 domains: patient assessment (1–8 points), teamwork (1–6 points), and treatment (1–4 points)], *TAS* Teamwork Assessment Scale [54], *TEAM* Team Emergency Assessment Measure [11 items across 3 domains using a 0–4 rating scale] [79], *TPA* team-assessment performance [3 domains: patient assessment (1–8 points), teamwork (1–6 points), and treatment (1–4 points)], *VAS* visual analogue scale

^a^ Data collection instruments developed by study authors

^b^ Origin of data collection instruments unclear

### Learner characteristics

In total, the 18 studies recruited 2459 learners. Of these, the majority were undergraduate students of varying professional backgrounds: 1814 nursing, 210 medical, 158 dental, 73 occupational therapy, and 39 physiotherapy students. Only 165 learners were postgraduate professionals: 129 doctors and 26 nurses. In all but four studies [15, 16, 49, 54], learners worked with their own professional group rather than as part of an interprofessional team.

### Self-led debriefing format

The specific debriefing activities, whether SLDs, FLDs or a combination of both, took several different formats and lasted between 3 and 90 min. Most SLDs utilised a written framework or checklist to guide learners through the debriefing, although this was unclear in two studies [42, 44]. Two studies required learners to independently self-reflect, via a written task, prior to commencing group discussion [49, 50]. Some studies included video playback within their debriefings [15, 16, 42, 43, 49–52].

### Data collection instruments and outcome measures

In total, 38 different data collection instruments were used across the 18 studies. These are listed along with their components and incorporated scales if described in sufficient detail within the primary study (Table 4). The validity and reliability of these instruments is variable. Indeed, 13 data collection instruments were developed by study authors without data on validity or reliability. Authors used one or more instruments to measure outcomes in five key domains (Table 5).

**Table 5:** Outcome measures

I. Debriefing quality (assessed either by learners themselves or by observers)	
II. Individual or group performance or competence (assessed by observers rating skills, knowledge, or behaviours)	
III. Learner self-confidence or self-assessed competence covering a range of skills and behaviours	
IV. Learner satisfaction or experience with the simulation or debriefing modality	
V. Debriefing content via qualitative data analysis using a constant comparison method	

### Key reported findings of studies

There was significant heterogeneity between the designs, aims, samples, SLD format, outcome measures and contexts of the 18 studies, with often conflicting and inherently biased findings due to study designs and outcome measures used. Nine studies reported equivalent outcomes regarding some elements of either debriefing quality, participant performance or competence, self-confidence or self-assessment of competence and participant satisfaction [15, 45–49, 52, 53, 56]. However, of these nine, five also reported that SLDs were significantly less effective if using other elements of the outcome measures [45, 46, 49, 52, 56]. In addition to these five, two studies reported decreased effectiveness of SLDs in comparison to FLDs or a combination of SLD + FLD [43, 50]. Conversely, only Lee et al. [52] and Oikawa et al. [48] reported any significant improvements with selected outcome measures with SLDs compared with FLDs, whilst Kündig et al. [47] reported improvements in two performance parameters with SLDs when compared with no debriefing.

Four studies investigated using a combination strategy of SLD + FLD and demonstrated either significantly improved or equivalent outcomes compared with either SLDs or FLDs only [49–51, 56]. Kang and Yu [51] reported significantly improved outcomes for problem-solving and debriefing satisfaction, but no differences in debriefing quality or team effectiveness. Other studies reported the opposite with significantly improved team effectiveness and debriefing quality, but no improvements in problem-solving or debriefing experience [49, 50]. Tutticci et al. [56] reported both significant and non-significant improvements in reflective thinking, dependent on which scoring tool was used. These findings, however, are in the context of variable quality appraisal scores (Table 3), wide variation in SLD formats and data collection instruments, and improved outcomes regardless of the method of debriefing used.

### Thematic analysis results

**Fig. 2:**
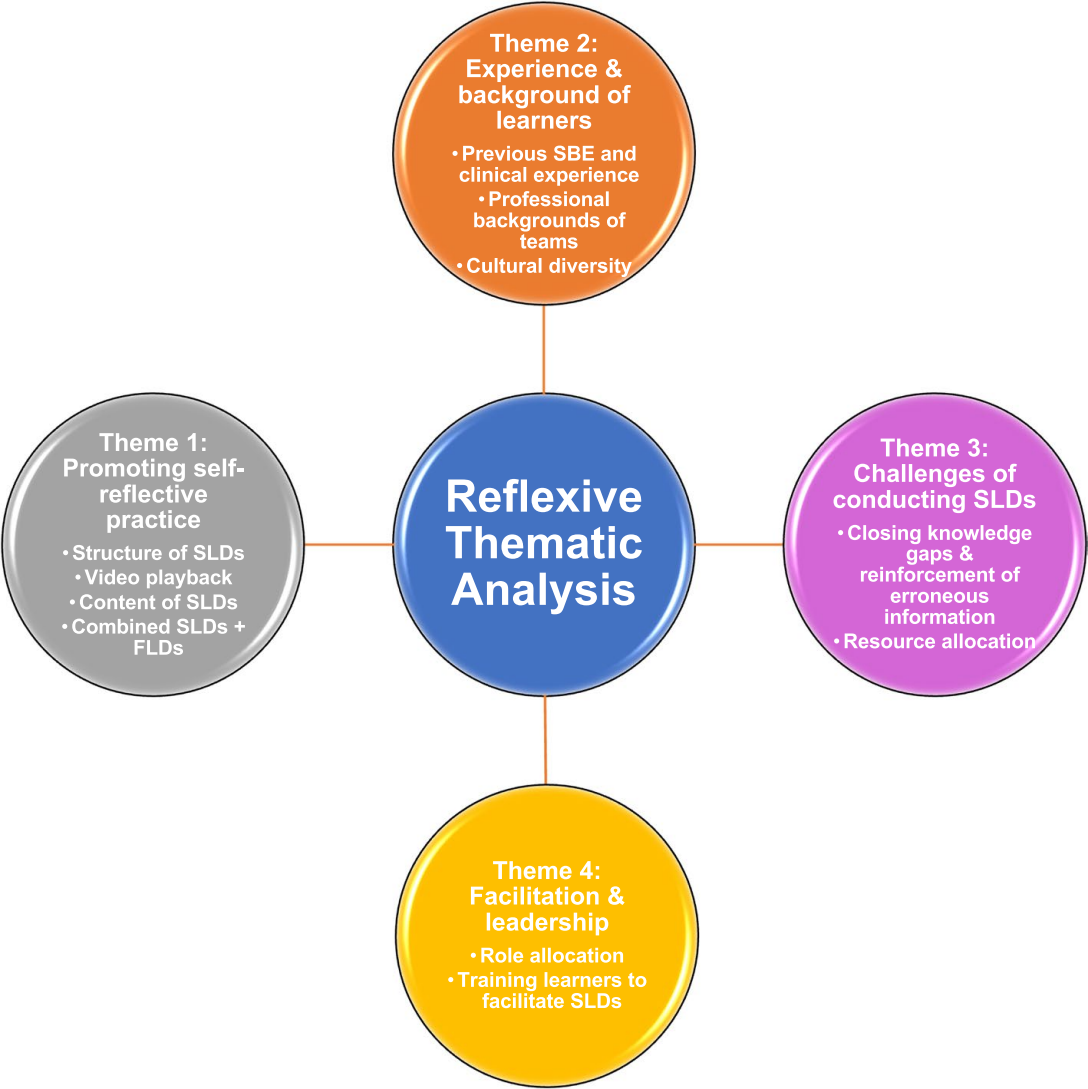
Thematic analysis map illustrating themes and subthemes

We undertook reflexive thematic analysis (RTA) of the data set, revealing four themes and 11 subthemes (Fig. 2). The process of tabulating themes and an exemplar of coding strategy and theme development can be found in Additional files 3 and 4.

#### Theme 1: Promoting self-reflective practice

The analysis of data revealed that promoting self-reflective practice is the most fundamental component of how and why SLDs influence debriefing outcomes. Debriefings can encourage groups of learners to critically reflect on their shared simulated experiences leading to enhanced cognitive, social, behavioural and technical learning [15, 16, 42, 43, 45–48, 50, 51, 53, 54, 56, 57]. Different components within SLDs, including structured frameworks, video playback, and debriefing content, may influence such self-reflective practice. Most authors advocated a printed framework or checklist to help guide learners through the SLD process. However, despite this, SLDs were found to be less structured than FLDs [16]. The Gather-Analyse-Summarise framework [59] was most commonly used [46, 49–51, 53]. One study compared two locally developed debriefing instruments, the Team Assessment Scales (TAS) and Quick-TAS (Q-TAS), concluding that the Q-TAS was more effective in enabling the analysis of actions, but equivalent in all other measures [54].

Video playback offered a form of feedback for learners that encouraged reflective processing of scenarios [15, 16, 52]. One article concluded quoting a learner: ‘I learned it’s worthwhile to revisit situations like this. I know I won’t always have video to critique, but being able to rethink through the appointment will be helpful to review which tactics helped and which ones need to be revised’ ([42], p., 929). In such a manner, video playback enables learners to perceive behaviours of which they were previously unaware [15]. Whilst many studies lacked interrogation of content within SLDs, Boet et al. [16] provided an extensive analysis, reporting that interprofessional SLDs centred on content such as situational awareness, leadership, communication, roles, and responsibilities. Furthermore, it was through learners’ perceived performance of this content that offered entry points into reflection [16]. Some studies required learners to document their thoughts and impressions [44, 45, 47, 48, 50, 53]. However, the influence of content documentation on promoting self-reflective practice was inconclusive.

Combined SLD + FLD strategies involved learner and faculty co-debriefing [56], or SLDs preceding FLDs [49–51]. Using the Reflective Thinking Instrument one study reported FLD and combined SLD + FLD groups demonstrated significantly higher levels of reflective thinking amongst learners compared with SLD groups [56]. Within the limitations of a tool with poor validity and reliability, this study provides the best evidence that a combination approach to debriefing groups may be the most beneficial method for encouraging learner critical self-reflection. This finding is supported by results from three other studies showing improved outcomes with combined debriefing strategies, across team effectiveness [49], debriefing quality [50], problem-solving processes [51] and satisfaction with debriefing [50, 51].

#### Theme 2: Experience and background of learners

The experience and background of learners has a profound impact on how and why SLDs influence debriefing outcomes. Previous SBE experience may significantly impact the ability of learners to meaningfully engage with the SLD process and influences their expectations as to how a simulated scenario will progress [15, 16]. Furthermore, previous experience with FLDs may positively contribute to rich reflective discussion within SLDs as learners are better placed to integrate FLD goals and processes within a new context [16]. Whilst its influence on the conduct of SLDs is less clear, Boet et al. [16] note that real-world clinical experience allows learners to recontextualise their simulated experiences more readily and may therefore act as an entry point into the reflective process. In teams from the same professional background, learners appreciated the value of learning from constructive exchanges of opinion between colleagues operating at the same level [42, 44, 45], and role-modelling teamwork behaviours [48], whilst interprofessional SLDs may help break down traditional working silos, and support learning in contexts that replicate clinical practice [15]. Finally, learners originated mainly from either South Korea or North America. Cultural differences between Korean and Western learners may affect debriefing practices, with Korean students being described as less expressive than their Western colleagues [46]. The impact of cultural diversity on SLD methods, however, was not specifically investigated [44, 46, 53].

#### Theme 3: Challenges of conducting SLDs

Challenges of conducting SLDs were constructed from the dataset, including closing knowledge gaps, reinforcement of erroneous information, and resource allocation. The absence of expert facilitators may present a missed learning opportunity, whereby erroneous information could be discussed and consolidated, thus negatively affecting subsequent performance [44, 45, 47, 51] and potentially persisting into clinical practice [46]. There was consistent student preference for FLDs over SLDs which may indicate learners seeking expert reassurance and accurate debriefing content not readily available from peers [43, 50]. By reducing the requirement for expensive faculty presence, a significant motivating factor for investigating and employing SLDs is the potential for reducing costs [15, 16, 44–46, 49, 57]. However, SLDs do not appear to negate the need for faculty presence completely, but rather limit their role for specific elements within a SLE [15, 16]. Furthermore, the most influential impact on debriefing outcomes may be the incorporation of SLDs in combination with, rather than at the expense of, FLDs [49–51, 56]. Finally, most articles integrated a FLD-element within their SLE, thereby negating positive impacts on resource allocation [15, 16, 42–46, 49–51, 54–57].

#### Theme 4: Facilitation and leadership

The facilitation and leadership of SLDs may have a considerable impact on how and why SLDs influence debriefing outcomes. Only five articles described how learners were allocated as leaders and facilitators of SLDs [43, 54–57]. Random allocation of learners to lead and facilitate SLDs occurred either prior to, or on the day of the SLE [54–56]. In two studies, learners took turns leading the debrief such that all learners facilitated at least one SLD [43, 57]. No articles discussed the influence of leadership and facilitation on learners, nor the learners’ reactions, thoughts, or feelings towards the role or the content and reflective learning with subsequent debriefings. In two articles describing the same learner sample, only one of 17 interprofessional SLDs was nurse-led, all others being led by a medical professional [15, 16]. Such situations may have unintended implications by reinforcing stereotypes and hierarchical power imbalances.

Learners were trained to lead the SLDs in only two studies. In one, learners were randomly allocated to lead the SLDs, and were directed to online resources, including videos, checklists, and relevant articles, to help prepare for this role prior to the SLE [56]. No information concerning learners’ engagement with the resources was documented. In another study, learners were given 60 min training on providing constructive feedback to peers, which did not lead to improved outcomes for debriefing quality, performance, or self-confidence [43].

## Discussion

The aim of this IR was to collate, synthesise and analyse the relevant literature to explore, with comparison to FLDs, how and why in-person SLDs influence debriefing outcomes for groups of learners in immersive SBE. The review identified 18 empirical studies with significant heterogeneity in respect to designs, contexts, learner characteristics, and data collection instruments. It is important to recognise that the review’s findings are limited by the variety and variability in quality of the data collection instruments and debriefing outcome measures used in these studies, as well as by some of the study designs themselves. Nevertheless, the findings of this review suggest that, across a range of debriefing outcomes, in situations where resources for FLDs are limited, SLDs can provide an alternative opportunity to safeguard effective learning. In some cultural and professional contexts, and for certain debriefing outcome measures, SLDs and FLDs may provide equivalent educational outcomes. Additionally, a small cohort of studies suggest that combined SLD + FLD strategies may be the optimal approach. Furthermore, SLDs influence debriefing outcomes most powerfully by promoting self-reflection amongst groups of learners.

### Promoting self-reflection

Aligned with social constructivist theory [80], the social interaction of collaborative group learning in a reflective manner can lead to the construction, promotion and sharing of a wide ranging of interpersonal and team-based skills [81, 82]. Currently, there is a lack of evidence concerning which frameworks are best suited to maximise such reflection [10], especially in SLDs. Whilst framework use is associated with improvements in debriefing quality and subsequent performance, some evidence suggests that, in terms of promoting reflective practice, the specific framework itself is of less importance than the skills of the facilitator using it and the context in which it is applied [7, 9, 10]. In SLDs, there is no facilitator to guide this process, and as such, one may infer that the framework itself may have relatively more influence on debriefing outcomes and the reflective process of learners when compared with their use in FLDs. Conversely, whichever framework is used, the quality of the SLDs were rated highly, implying that it may be the structure provided by the framework, as opposed to the framework content, that is the critical factor for promoting reflection. Based on the findings of their qualitative study in which self-reflexivity, connectedness and social context informed learning within debriefings, Gum et al. [83] developed a reflective conceptual framework rooted in transformative learning theory [84], which purported to enable learners to engage in critical discourse and learning. By placing learners at the centre of their model, and by focusing on the three themes previously mentioned, this framework seems suited to groups of learners in SLDs. However, like many other debriefing frameworks, it remains untested in SLD contexts. In a study of business students, Eddy et al. [85] describe using an online tool that captured and analysed individual team members’ perceptions of an experience anonymously. The tool then prioritised reported themes to create a customised guide for teams to use in a subsequent in-person group SLD. The study reported that using this tool resulted in superior team processes and subsequent greater team performance when compared to SLDs using a generic debriefing guide only. Such tools may have a place in promoting self-reflection in healthcare SBE, such as with postgraduate learners with previous experiences of debriefings or those who have undertaken training in debriefing facilitation.

Furthermore, other structures or techniques that may help influence and promote self-reflection amongst groups of learners in SLDs are, as yet, untested in this context. For example, SLDs could take the form of in-person or online post-scenario reflective activities, in which learners work collaboratively on pre-determined tasks that align to ILOs. Examples such as escape room activities in SBE, in which learners work together to solve puzzles and complete tasks through gamified scenarios, have used concepts grounded in self-determination theory [86], with promising results in terms of improving self-reflection and learning outcomes [87, 88]. Meanwhile, individual virtual SLD interventions, rooted in Kolb’s experiential learning theory [89], have been tested and purport to enable critical reflection amongst users [90, 91]. Whilst such approaches may be relatively resource-intensive to create, they could be applied to SLDs for groups of learners in immersive SBE and prove resource-efficient once established.

### Video playback

In both individual and group SLD exercises, video playback can allow learners to self-reflect, analyse performance, minimise hindsight bias, and identify mannerisms or interpersonal behaviours that may otherwise remain hidden [15, 42, 52, 92–95]. These findings are supported by situated learning theory whereby learning can be associated with repeated cycles of visualisation of, and engagement with, social interactions and interpersonal relationships which enable co-construction of knowledge amongst learners [96]. Conversely, in group SLD contexts, watching video playback may have unintended consequences for psychological safety, making learners feel self-conscious and anxious, and impact negatively on their ability to meaningfully engage with reflective learning [93]. A systematic review concluded that the benefits of video playback are highly dependent on the skill of the facilitator rather than the video playback itself [95], and as such its role influencing debriefing outcomes in SLDs remains uncertain.

### Combining self-led and facilitator-led debriefings

The findings of this review suggest that employing combinations of SLDs and FLDs may optimise participant learning [49–51, 56], whilst acknowledging that this may also be dependent on other variables such as the expertise of debriefers and contexts within which debriefings occur. Whilst the reported improved outcomes are situated in the context of in-person SLDs for groups of learners, they are supported by the wider literature. For example, a Canadian research group investigated combined in-person and virtual individual SLD formats with FLDs, reporting improved debriefing outcomes across multiple domains including knowledge gains, self-efficacy, maximising reflection, and debriefing experience [90, 97–99]. SLD components of the combined strategy enable learners to reflect, build confidence, identify knowledge gaps, collect, and organise their thoughts and prepare for group interaction prior to a FLD [90, 97–99]. However, limitations of these studies include the unreliability of outcome measures.

### Facilitation and leadership

Only two studies provided training for learners in how to facilitate debriefings and provide constructive feedback [43, 56]. This is surprising given the emphasis of faculty development in the SBE literature [6, 9, 28, 100]. RTA of the data highlighted how the potential influence of previous experience with FLDs may influence learners’ ability to actively engage in the reflective nature of the SLD process [15, 16]. This brings into question whether learners should have some familiarity of debriefing processes, either via previous experience or targeted training, prior to facilitating group SLDs.

Variables such as learners’ debriefing experiences and educational context have implications for the interpretation of the findings of this review. Having previous experience with FLDs may potentially influence learners’ abilities to actively engage in the reflective nature of the SLD process [15, 16] bringing into question whether learners should have some familiarity of debriefing processes, either via prior experience or targeted training, prior to being expected to facilitate or lead a group SLD. This further raises questions about whether SLDs may or may not be more suitable for certain populations, such as students undergoing early training or postgraduates who are relatively more experienced in SBE. Training peers as facilitators, who then act in an ‘instructor’ role, rather than as part of the learner group, has also been reported as an effective method to positively influence debriefing outcomes [101, 102]. However, training learners to facilitate SLDs involves significant resource commitments, thus negating some of the initial reasons for instigating SLDs.

### Data collection instruments and outcome measures

The studies included in this review used multiple data collection tools to gauge the influence of SLDs on debriefing outcomes across five domains (Table 5). The diversity in approaches to outcome measurement is problematic as it impedes the ability to compare studies fairly, effectively, and robustly [103]. Certain instruments, such as the Debriefing Assessment for Simulation in Healthcare- Student Version [68] and the Debriefing Experience Scale [69], are validated and reliable tools for assessing learner perceptions of, and feelings towards, debriefing quality in certain contexts. However, learner perceptions of debriefing quality do not necessarily translate to objective evaluation of debriefing practices. Additionally, some studies relied on learner self-confidence and self-reported assessment questionnaires for their outcome measures, despite self-perceived competence and confidence being a poor surrogate marker for clinical competence [104]. Commonly used tools measuring debriefing quality may not be suitable for SLDs and having a ‘one-size-fits-all’ approach could invalidate results [105]. To our knowledge, there is no validated or reliable tool currently available that specifically assesses the debriefing quality of SLDs.

### Psychological safety

One important challenge of conducting SLDs, which was not constructed through the RTA of this dataset, is ensuring psychological safety of learners in debriefings. Psychological safety is defined as ‘a shared belief held by members of a team that the team is safe for interpersonal risk taking’ ([106], p., 350) and its establishment, maintenance and restoration in debriefings is of paramount importance for learners participating in SBE [107, 108]. Oikawa et al. [48] stated that ‘self-debriefing may augment reflection through the establishment of an inherently safe environment’ ([48], p., 130), although how safe environments are ‘inherent’ within SLDs is unclear. Tutticci et al. [56] quote secondary sources [83, 109] stating that peer groups can improve collegial relationships and engender safe learning environments that improve empathy whilst reducing the risk of judgement. Conversely, it may also transpire that psychologically unsafe environments are fostered, leading to unintended harmful practices. In interprofessional contexts where historical power imbalances, hierarchies and professional divisions can exist [11, 110, 111], and in which facilitator skill has been the most frequently cited enabler of psychological safety [112], one can infer that threats to psychological safety may be accentuated in SLDs.

In contrast, researchers found the process of engaging in an individual SLD enhanced psychological safety by helping learners decrease their stress and anxiety, thus leading to more active engagement and meaningful dialogue in subsequent FLDs [99]. Another study reported learners describing the familiarity of connecting with known peers within SLDs fostered psychological safety and enabled learning [98]. However, these studies were excluded from this review due to having individual rather than group SLDs. Nevertheless, their findings that combined SLD + FLD strategies enable psychological safety may partially explain the findings of this review, and psychological safety may therefore be a central concept in understanding how and why SLDs influence debriefing outcomes.

For teams regularly working together in clinical contexts, their antecedent psychological safety has a major influence on any SLEs they undertake [113]. This subsequently impacts on how team members, both individually and collectively, experience psychological safety within their real clinical environment [113]. The place of SLDs in such contexts, along with their potential advantages and risks, remains undetermined.

### Limitations

This review specifically investigates in-person group debriefings, and therefore, the results may not be applicable to individual or virtual SLD contexts. The inclusion criteria allowed for published peer-reviewed empirical research studies in English, excluding grey literature. This may introduce bias with some evidence suggesting that excluding grey literature can lead to over-exaggerated conclusions [114, 115], and concerns regarding publishing bias [116]. We also acknowledge that the choices made in constructing and implementing our search strategy (Additional file 1) may have impacted the total number of articles identified for inclusion in this review. Finally, the heterogeneity of the included studies limits the certainty with which generalisable conclusions can be made. Conversely, heterogeneity enables a diverse body of evidence to be analysed and better informs the need for future research and where gaps may lie.

### Recommendations for future research

The findings of this review have highlighted several areas requiring further research. Firstly, the role of combining group SLDs with FLDs should be explored, both quantitatively and qualitatively, to explain its place within immersive SBE. Secondly, to inform best practice, different methods, structures and frameworks of group SLDs need investigating to assess what may work, for whom and in which context. This extends to further research investigating different groups, such as interprofessional learners, to ascertain if certain contexts are more suitable for SLDs than others. Such work may feed into the production of guidelines to help standardise SLD practices across these differing contexts. Thirdly, assessment and testing of data collection instruments is required, as current tools are not fit for purpose. Clarification of what is suitable and measurable in terms of debriefing quality and learning outcomes, especially in relation to group SLDs, is needed. Finally, whilst research into fostering psychological safety in FLDs is emerging, the same is not true in the context of SLDs and this needs to be explored to ensure that SLDs are not psychologically harmful for learners.

## Conclusions

To our knowledge this is the first review to explore how and why in-person SLDs influence debriefing outcomes for groups of learners in immersive SBE. The findings address an important gap in the literature and have significant implications for simulation-based educators involved with group debriefings across a variety of contexts. The synthesised findings of this review suggest that, across a range of debriefing outcome measures, in-person SLDs for groups of learners following immersive SBE are preferable to conducting no debriefing at all. In certain cultural and professional contexts, such as postgraduate learners and those with previous debriefing experience, SLDs can support effective learning and may provide equivalent educational outcomes to FLDs or SLD + FLD combination strategies. Furthermore, there is some evidence to suggest that SLD + FLD combination approaches may optimise participant learning, with this approach warranting further research.

Under certain conditions and circumstances, SLDs can enable learners to achieve suitable levels of critical self-reflection and learning. Similar to FLDs, promoting self-reflective practice within groups of learners is the fundamental method of how and why SLDs influence debriefing outcomes because it is through this metacognitive skill that effective learning and behavioural consolidation or change can occur. However, more work is required to ascertain for whom and in what contexts SLDs may be most appropriate. In situations where resources for FLDs are limited, SLDs may provide an alternative opportunity to enable effective learning. However, their true value within the scope of immersive SBE may lie as an adjunctive method alongside FLDs.

### Supplementary information

**Additional file 1.** Final search strategies of electronic bibliographic databases.

**Additional file 2.** List of studies identified for full-text screening and reasons for exclusion.

**Additional file 3.** Tabulating themes developed via reflexive thematic analysis.

**Additional file 4.** Exemplar of coding strategy and theme development.

## Data Availability

The datasets used and/or analysed during the current study are available within the article and supplementary files or from the corresponding author on reasonable request.
