# Supplementary material for: Exploring in-person self-led debriefings for groups of learners in simulation-based education: an integrative review
Source: Adv Simul (Lond). 2024 Jan 16;9:5. doi: 10.1186/s41077-023-00274-z (PMC10790376; doi:10.1186/s41077-023-00274-z)
Supplement: Supplementary file 1 — Additional file 1. Final search strategies of electronic bibliographic databases. [file 41077_2023_274_MOESM1_ESM.docx]

### Additional File 1: Final search strategies of electronic bibliographic databases (conducted on 14/10/2022)

To help develop our search strategies, we modified the PICOS (population, intervention/interest, comparison, outcome, study design) [32] framework by supplementing the ‘comparison’ arm with ‘context’ as described by Dhollande et al. [33]. This suited our study in which the research question of how and why SLDs influence debriefing outcomes, is situated within the context of well-established FLD practices within SBE. By including FLDs and associated synonyms as search terms within the strategy, we have minimised the risk that the search overlooks studies that described SLDs as what they are not- i.e. ‘without an facilitator’. Conversely however, we accept that this search strategy may have therefore retrieved more non-relevant studies investigating only FLDs. In contrast, to reduce the number of non-relevant articles in the search string, we decided against truncating ‘self-led’, ‘self-directed’, ‘self-guided’, ‘self-facilitated’ and ‘self-debrief*’ to ‘self’ alone, as articles retrieved would have included terms such as self-discovery, self-esteem, and self-discipline. Whilst acknowledging that this may seem at odds with our previous approach of including the term FLDs and associated synonyms, we argue that the widespread established practice of FLDs in healthcare simulation warranted this decision. Furthermore, we chose to forgo specifying outcome terms and study designs, as by definition IRs encourage the incorporation of diverse study methodologies [30].

We employed keywords, free-text words, and index terms of relevant articles retrieved during a preliminary scoping search of the literature into pilot iterative searches of the PubMed database. Based on preliminary results of these searches and subsequent refinement of terms, we formulated a list of final relevant, inclusive, and precise search terms. These were subsequently used in the final customised searches of the seven databases included in this study. To help develop appropriate search strings within PubMed, the Medical Subject Heading (MeSH) of ‘simulation training’ was incorporated. Conversely however, ‘high fidelity simulation training’ was omitted as its inclusion did not result in an expansion of returns. Furthermore, certain key terms, such as ‘simulation’ and ‘debriefing’, were truncated to their stem words (e.g. simul*), thus ensuring that the diverse terminology used across health professions education literature was accounted for within the results. Terms were then combined using the ‘OR’ Boolean operator to maximise sensitivity within each category. To enhance the comprehensiveness of the search, we amalgamated the intervention and compactor/context groups with the ‘OR’ Boolean operator. The results for each category were subsequently combined using the ‘AND’ Boolean operator, ensuring that components of each category were represented in the final search results. The search strategies were altered to accommodate the characteristics of each specific database and appropriate limits, such as language, were applied that this stage. A full delineation of each search strategy is presented below.

In the interests of transparency, we acknowledge that the choices made here in implementing these search strategies may have impacted the total number of articles identified.

1. PubMed

| #1 | **Simulation training [MeSH] OR simulation-based OR simulation-enhanced OR "simulation training" OR "simulation teaching" OR "simulation event" OR (immersion AND simulation)** | 26,658 |
| --- | --- | --- |
| #2 | **(Facilitator-led OR Instructor-led OR Faculty-led OR "Instructor debrief*" OR Facilitated) OR (Search: Self-led OR Peer-led OR Group-led OR Participant-led OR Student-led OR Self-directed OR Student-directed OR Self-guided OR Self-facilitated OR Peer-facilitated OR Group-facilitated OR Student-facilitated OR Self-debrief* OR Peer-debrief* OR Group-debrief* OR "Self debrief*" OR "Peer debrief*" OR "Group debrief*" OR Within-team)** | 659,522 |
| #3 | **Debrief* OR Conversation*** | 31,513 |
| #4 | (#2 AND #3): **((Facilitator-led OR Instructor-led OR Faculty-led OR "Instructor debrief*" OR Facilitated) OR (Search: Self-led OR Peer-led OR Group-led OR Participant-led OR Student-led OR Self-directed OR Student-directed OR Self-guided OR Self-facilitated OR Peer-facilitated OR Group-facilitated OR Student-facilitated OR Self-debrief* OR Peer-debrief* OR Group-debrief* OR "Self debrief*" OR "Peer debrief*" OR "Group debrief*" OR Within-team)) AND (Debrief* OR Conversation*)** | 3,795 |
| #5 | (#1 AND #4): **(((Facilitator-led OR Instructor-led OR Faculty-led OR "Instructor debrief*" OR Facilitated) OR (Search: Self-led OR Peer-led OR Group-led OR Participant-led OR Student-led OR Self-directed OR Student-directed OR Self-guided OR Self-facilitated OR Peer-facilitated OR Group-facilitated OR Student-facilitated OR Self-debrief* OR Peer-debrief* OR Group-debrief* OR "Self debrief*" OR "Peer debrief*" OR "Group debrief*" OR Within-team)) AND (Debrief* OR Conversation*)) AND (Simulation training [MeSH] OR simulation-based OR simulation-enhanced OR "simulation training" OR "simulation teaching" OR "simulation event" OR (immersion AND simulation))** | 381 |

1. CENTRAL (Cochrane Central Register of Controlled Trials)

| #1 | MeSH descriptor: [Simulation Training] explode all trees | 1181 |
| --- | --- | --- |
| #2 | Simul* OR Simulation-based OR simulation-enhanced OR "simulation training" OR "simulation teaching" OR "simulation event" OR (immersion AND simulation) | 42,756 |
| #3 | (Facilitator-led OR Instructor-led OR Faculty-led OR "Instructor debrief*" OR Facilitated) OR (Self-led OR Peer-led OR Group-led OR Participant-led OR Student-led OR Self-directed OR Student-directed OR Self-guided OR Self-facilitated OR Peer-facilitated OR Group-facilitated OR Student-facilitated OR Self-debrief* OR Peer-debrief* OR Group-debrief* OR "Self debrief*" OR "Peer debrief*" OR "Group debrief*" OR Within-team) | 9,549 |
| #4 | Debrief* OR Conversation* | 3,943 |
| #5 | #1 AND #2 AND #3 AND #4 | 12 |
| #6 | #2 AND #3 AND #4 | 93 |

1. EMBASE

| #1 | Simulation-based OR simulation-enhanced OR "simulation training" OR "simulation teaching" OR "simulation event" OR (immersion AND simulation) | 20,001 |
| --- | --- | --- |
| #2 | (Facilitator-led OR Instructor-led OR Faculty-led OR "Instructor debrief*" OR Facilitated) OR (Self-led OR Peer-led OR Group-led OR Participant-led OR Student-led OR Self-directed OR Student-directed OR Self-guided OR Self-facilitated OR Peer-facilitated OR Group-facilitated OR Student-facilitated OR Self-debrief* OR Peer-debrief* OR Group-debrief* OR "Self debrief*" OR "Peer debrief*" OR "Group debrief*" OR Within-team) | 146,673 |
| #3 | Debrief* OR Conversation* | 46,297 |
| #4 | #2 AND #3 | 2,016 |
| #5 | #4 AND #1 | 251 |

1. ERIC

| #1 | Simul* OR Simulation-based OR simulation-enhanced OR "simulation training" OR "simulation teaching" OR "simulation event" OR (immersion AND simulation) | 42,611 |
| --- | --- | --- |
| #2 | (Facilitator-led OR Instructor-led OR Faculty-led OR "Instructor debrief*" OR Facilitated) OR (Self-led OR Peer-led OR Group-led OR Participant-led OR Student-led OR Self-directed OR Student-directed OR Self-guided OR Self-facilitated OR Peer-facilitated OR Group-facilitated OR Student-facilitated OR Self-debrief* OR Peer-debrief* OR Group-debrief* OR "Self debrief*" OR "Peer debrief*" OR "Group debrief*" OR Within-team) | 15,835 |
| #3 | Debrief* OR Conversation* | 23,571 |
| #4 | #2 AND #3 | 591 |
| #5 | #4 AND #1 | 34 |

1. SCOPUS

| #1 | (((facilitator-led  OR instructor-led  OR  faculty-led  OR  "Instructor debrief*"  OR  facilitated)  OR  (search:  AND self-led  OR  peer-led  OR  group-led  OR  participant-led  OR  student-led  OR  self-directed  OR  student-directed  OR  self-guided  OR  self-facilitated  OR  peer-facilitated  OR  group-facilitated  OR  student-facilitated  OR  self-debrief*  OR  peer-debrief*  OR  group-debrief*  OR  "Self debrief*"  OR  "Peer debrief*"  OR  "Group debrief*"  OR  within-team)) AND (debrief*  OR  conversation*)) AND (simulation AND training AND  [mesh]  OR  simulation-based  OR  simulation-enhanced  OR  "simulation training"  OR  "simulation teaching"  OR  "simulation event"  OR (immersion AND simulation)) AND (LIMIT-TO ( DOCTYPE “ar” ) OR LIMIT-TO (DOCTYPE, “sh”)) AND ( LIMIT-TO (LANGUAGE, “English” )) AND ( EXCLUDE ( SUBJAREA, MATH” ) OR EXCLUDE ( SUBJAREA, “COMP” ) OR EXCLUDE (SUBJAREA, “BUSI” ) OR EXCLUDE ( SUBJAREA, “ENGI” )) | 358 |
| --- | --- | --- |

1. CINAHL Plus

| #1 | Simul* OR Simulation-based OR simulation-enhanced OR "simulation training" OR "simulation teaching" OR "simulation event" OR (immersion AND simulation) | 128,861 |
| --- | --- | --- |
| #2 | (Facilitator-led OR Instructor-led OR Faculty-led OR "Instructor debrief*" OR Facilitated) OR (Self-led OR Peer-led OR Group-led OR Participant-led OR Student-led OR Self-directed OR Student-directed OR Self-guided OR Self-facilitated OR Peer-facilitated OR Group-facilitated OR Student-facilitated OR Self-debrief* OR Peer-debrief* OR Group-debrief* OR "Self debrief*" OR "Peer debrief*" OR "Group debrief*" OR Within-team) | 27,336 |
| #3 | Debrief* OR Conversation* | 25,601 |
| #4 | #2 AND #3 | 868 |
| #5 | #4 AND #1 | 169 |

1. PsycINFO

| #1 | Simulation-based OR simulation-enhanced OR "simulation training" OR "simulation teaching" OR "simulation event" OR (immersion AND simulation) | 2,021 |
| --- | --- | --- |
| #2 | (Facilitator-led OR Instructor-led OR Faculty-led OR "Instructor debrief*" OR Facilitated) OR (Self-led OR Peer-led OR Group-led OR Participant-led OR Student-led OR Self-directed OR Student-directed OR Self-guided OR Self-facilitated OR Peer-facilitated OR Group-facilitated OR Student-facilitated OR Self-debrief* OR Peer-debrief* OR Group-debrief* OR "Self debrief*" OR "Peer debrief*" OR "Group debrief*" OR Within-team) | 35,091 |
| #3 | Debrief* OR Conversation* | 45,199 |
| #4 | #2 AND #3 | 1,112 |
| #5 | #4 AND #1 | 15 |
