# Supplementary material for: Exploring in-person self-led debriefings for groups of learners in simulation-based education: an integrative review
Source: Adv Simul (Lond). 2024 Jan 16;9:5. doi: 10.1186/s41077-023-00274-z (PMC10790376; doi:10.1186/s41077-023-00274-z)
Supplement: Supplementary file 2 — Additional file 2. List of studies identified for full-text screening and reasons for exclusion. [file 41077_2023_274_MOESM2_ESM.docx]

### Additional File 2: List of studies identified for full-text screening and reasons for exclusion

| **Studies Identified for Full-text Screening via Database Search** | **Included/Excluded** | **Reasons For Exclusion** |
| --- | --- | --- |
| Andrews, E., et al. (2019). Comparison of dental students’ perceived value of faculty vs. peer feedback on non-technical clinical competency assessments. Journal of Dental Education. 83(**5**): 536-545 | Included | Not applicable |
| Bae, J., et al. (2019). Development of simulation education debriefing protocol with faculty guide for enhancement clinical reasoning. BMC Medical Education. 19(**1**) | Excluded | Study describes FLDs exclusively or compares FLDs to no debriefing |
| Black, S. E. (2018). Obstetric emergencies: Enhancing the multidisciplinary team through simulation. British Journal of Midwifery. 26(**2**): 96-102. | Excluded | Study describes FLDs exclusively or compares FLDs to no debriefing |
| Boet, S., et al. (2011). Looking in the mirror: self-debriefing versus instructor debriefing for simulated crises. Critical Care Medicine. 39(**6**): 1377-1381. | Excluded | Debriefings only including one learner/participant |
| Boet, S., et al. (2013). Within-team debriefing versus instructor-led debriefing for simulation-based education: a randomized controlled trial. Annals of Surgery. 258(**1**): 53-58. | Included | Not applicable |
| Boet, S., et al. (2016). Interprofessional team debriefings with or without an instructor after a simulated crisis scenario: an exploratory case study. Journal of Interprofessional Care. 30(**6**): 717-725. | Included | Not applicable |
| Boet, S., et al. (2017). Debriefing decreases mental workload in surgical crisis: a randomized controlled trial. Surgery. 161(**5**): 1215-1220. | Excluded | Multiple exclusion criteria:   - Debriefings only including one learner/participant - Study describes FLDs exclusively or compares FLDs to no debriefing |
| Brown, J., et al. (2017). Exploring the use of student-led simulated practice learning in pre-registration nursing programmes. Nursing Standard. 32(**4**): 50-58. | Excluded | Study describes FLDs exclusively or compares FLDs to no debriefing |
| Bussard, M. E. (2016). Self-reflection of video-recorded high-fidelity simulations and development of clinical judgement. Journal of Nursing Education. 55(**9**): 522-527. | Excluded | Debriefings only including one learner/participant |
| Casler, K., et al. (2022). The effect of asynchronous group discussions on nurse practitioner student debriefing experience in virtual simulation. Journal of the American Association of Nurse Practitioners. 34(**7**): 901-908. | Excluded | Virtual/online/tele-simulation and debriefing study |
| Chen, W., et al. (2019). Using instrument-guided team reflection and debriefing to cultivate teamwork, knowledge, skills, and attitudes in pre-clerkship learning teams. Medical Science Educator. 29(**1**): 45-50. | Excluded | Non-immersive SLEs |
| Ciceron, F., et al. (2021). Individual versus collective debriefing after interprofessional training course simulation: the randomised DEBRIEF-SIM trial. Anaesthesia Critical Care and Pain Medicine. 40(**2**). | Excluded | Study describes FLDs exclusively or compares FLDs to no debriefing |
| Corbett, N., et al. (2012). Debriefing as a strategic tool for performance improvement. Journal of Obstetric, Gynecologic, and Neonatal Nursing. 41(**4**): 572-579. | Excluded | Clinical event debriefing |
| Corns, P., & Thomas, L. (2022). Remote-facilitated mental simulation to bridge the theory-practice divide. Journal of Paaramedic Practice. 14(**2**): 73-81. | Excluded | Virtual/online/tele-simulation and debriefing study |
| Curtis, E., et al. (2016). Incorporating peer-to-peer facilitation with a mid-level fidelity student led simulation experience for undergraduate nurses. Nurse Education in Practice. 20: 80-84. | Included | Not applicable |
| Dennis, D., et al. (2020). Can student-peers teach using simulated-based learning as well as faculty: a non-equivalent posttest-only study. Nurse Education Today. 91. | Excluded | Study describes FLDs exclusively or compares FLDs to no debriefing |
| Doherty-Restrepo, J., et al. (2018). Students’ perception of peer and faculty debriefing facilitators following simulation-based education. Journal of Allied Health. 47(**2**): 107-112. | Excluded | Study describes FLDs exclusively or compares FLDs to no debriefing |
| Edwards, S., et al. (2018). Student-led simulation: preparing students for leadership. Nursing Management. 25(**5**). | Excluded | Study describes FLDs exclusively or compares FLDs to no debriefing |
| Fan, H. J., et al. (2017). Effectiveness of hands-on cardiopulmonary resuscitation practice with self-debriefing for healthcare providers: a simulation-based controlled trial. Hong Kong Journal of Emergency Medicine. 24(**6**): 268-274. | Excluded | Debriefings only including one learner/participant |
| Fisher, J., et al. (2014). Hands on + hands free: simulated on-call interaction. Clinical Teacher. 11(6), 425-428. | Excluded | Non-immersive SLEs |
| Glatts, L., et al. (2021). Student perspectives of interprofessional group debriefing: use of the national league for nursing guide for teaching thinking. Nursing Education Perspectives. 42(**1**): 36-38. | Excluded | Study describes FLDs exclusively or compares FLDs to no debriefing |
| Goodwin, C. D. G., et al. (2021). Development of a novel and scalable simulation-based teamwork training model using within-group debriefing of observed video simulation. The Joint Commission Journal on Quality and Patient Safety. 47(**6**): 385-391. | Excluded | Multiple exclusion criteria:   - Non-immersive SLEs - Study describes FLDs exclusively or compares FLDs to no debriefing |
| Ha, E-H. (2020). Effects of peer-led debriefing using simulation with case-based learning: written vs. observed debriefing. Nurse Education Today. 84: 104249 | Included | Not applicable |
| Ha, E-H., & Lim, E. J. (2018). Peer-led written debriefing versus instructor-led oral debriefing: using multimode simulation. Clinical Simulation in Nursing. 18:38-46. | Included | Not applicable |
| Ha, E-H., & Song, H. S. (2015). The effects of structured self-debriefing using on the clinical competency, self-efficacy, and educational satisfaction in nursing students after simulation. The Journal of Korean Academic Society of Nursing Education. 21(**4**): 445-454. | Excluded | Study reported in a language other than English |
| Isaranuwatchai, W., et al. (2017). A cost-effectiveness analysis of self-debriefing versus instructor debriefing for simulated crises in perioperative medicine in Canada. Journal of Educational Evaluation for Health Professions. 13: 44. | Excluded | Debriefings only including one learner/participant |
| Kang, K., & Yu., M. (2018). Comparison of student self-debriefing versus instructor debriefing in nursing simulation: a quasi-experimental study. Nurse Education Today. 65: 67-73. | Included | Not applicable |
| Kim, S. S., & De Gagne, J. C. (2018). Instructor-led vs. peer-led debriefing in preoperative care simulation using standardized patients. Nurse Educator Today. 71: 34-39. | Included | Not applicable |
| Kun, Y., et al. (2019). Self-debriefing model based on an integrated video-capture system: an efficient solution to skill degradation. Journal of Surgical Education. 76(**2**): 362-369. | Excluded | Multiple exclusion criteria:   - Non-immersive SLEs - Debriefings only including one learner/participant |
| Kündig, P., et al. (2020). More than experience: a post-task reflection intervention among team members enhances performance in student teams confronted with a simulated resuscitation task-a prospective randomised trial. BMJ Simulation and Technology-Enhanced Learning. 6(**2**): 81-86. | Included | Not applicable |
| Lapum, J. L., et al. (2019). Self-debriefing in virtual simulation. Nurse Educator. 44(**6**). E6-E8. | Excluded | Multiple exclusion criteria:   - Non-empirical research - Debriefings only including one learner/participant - Virtual/online/tele-simulation and debriefing study |
| Lee, M. N., et al. (2020). Comparing the learning effects of debriefing modalities for the care of premature infants. Nursing and Health Sciences. 22(**2**): 243-253. | Included | Not applicable |
| Leigh, G. T., et al. (2017). A nurse educator’s guide to student-led debriefing. Teaching and learning in nursing. 12(**4**): 309-311. | Excluded | Multiple exclusion criteria:   - Non-empirical research - Study describes FLDs exclusively or compares FLDs to no debriefing |
| Luctkar-Flude, M., et al. (2017). Comparing instructor-led versus student-led simulation facilitation methods for novice nursing students. Clinical Simulation in Nursing. 13(**6**): 264-269. | Excluded | Study describes FLDs exclusively or compares FLDs to no debriefing |
| MacKenna, V., et al. (2021). Self-debriefing after virtual simulation: measuring depth of reflection. Clinical Simulation in Nursing. 52: 59-67. | Excluded | Debriefings only including one learner/participant |
| MacLean, S., et al. (2019). Video reflection in discharge communication skills training with simulated patients: a qualitative study of nursing students’ perceptions. Clinical Simulation in Nursing. 28: 15-24. | Excluded | Debriefings only including one learner/participant |
| Maneejak, N., & Yasri, P. (2019). NSMU: a reflection model for nursing students practicing with high fidelity simulation. International Journal of Innovation, Creativity and Change. 5(**2**): 54-66. | Excluded | Study describes FLDs exclusively or compares FLDs to no debriefing |
| Miller, S., & Miller, M. A. (2021). Mind the gap! A strategy to bridge the time between simulation and debriefing. Clinical Simulation in Nursing. 51: 10-13. | Excluded | Multiple exclusion criteria:   - Non-empirical research - Debriefings only including one learner/participant |
| Na, Y. H., & Roh, Y. S. (2021). Effects of peer-led debriefing on cognitive load, achievement emotions, and nursing performance. Clinical Simulation in Nursing. 55: 1-9. | Included | Not applicable |
| Nunnink, L., et al. (2021). Peer-assisted learning in simulation-based medical education: a mixed methods exploratory study. BMJ Simulation and Technology-Enhanced Learning. 7(**5**): 366-371. | Excluded | Study describes FLDs exclusively or compares FLDs to no debriefing |
| Oikawa, S., et al. (2016). Self-debriefing vs instructor debriefing in a pre-internship simulation curriculum: night on call. Hawaii Journal of Medicine and Public Health. 75(**5**): 127-132. | Included | Not applicable |
| Paige, J. T., et al. (2021). Improvement in student-led debriefing analysis after simulation-based team training using a revised teamwork assessment tool. Surgery. 170(**6**): 1659-1664. | Included | Not applicable |
| Philippon, A. L., et al. (2022). Innovating for a safe simulation challenge during a pandemic: an avatar simulation concept. Simulation in Healthcare. 17(**2**): 138-139. | Excluded | Debriefings only including one learner/participant |
| Presti, C. R., et al. (2020). Peer debriefing in simulation-based education. Nurse Educator. 45(**6**): 342. | Excluded | Multiple exclusion criteria:   - Non-empirical research - Grey literature - Non peer-reviewed - Study describes FLDs exclusively or compares FLDs to no debriefing |
| Roh, Y. S., et al. (2016). Comparison of instructor-led versus peer-led debriefing in nursing students. Nursing & Health Sciences. 18(**2**): 238-245. | Excluded | Non-immersive SLEs |
| Rueda-Medina, B., et al. (2020). A combination of self-debriefing and instructor-led debriefing improves team effectiveness in health science students. Nurse Educator. 46(**1**): E7-E11. | Included | Not applicable |
| Rueda-Medina, B., et al. (2021). Peer debriefing versus instructor-led debriefing for nursing simulation. Journal of Nursing Education. 60(**2**): 90-96. | Included | Not applicable |
| Ryoo, E. N., & Ha, E. (2015). The importance of debriefing in simulation-based learning: comparison between debriefing and no debriefing. Computers, Informatics, Nursing. 33(**12**): 538-545. | Excluded | Study describes FLDs exclusively or compares FLDs to no debriefing |
| Sukalich, S., et al. (2014). Teaching medical error disclosure to residents using patient-centered simulation training. Academic Medicine. 89(**1**): 136-143. | Excluded | Debriefings only including one learner/participant |
| Szyld, E. G., et al. (2021). Self-directed video versus instructor-based neonatal resuscitation training: a randomized controlled blinded non-inferiority multicenter international study. Journal of Perinatology. 41: 1583-1589. | Excluded | Non-immersive SLEs |
| Tudor, G. J., et al. (2020). The equivalence of video self-review versus debriefing after simulation: can faculty resources be reallocated? AEM Education and Training. 4(**1**): 36-42. | Excluded | Study describes FLDs exclusively or compares FLDs to no debriefing |
| Tutticci, N., et al. (2017). Student facilitation of simulation debrief: measuring reflective thinking and self-efficacy. Teaching & Learning in Nursing. 12(**2**): 128-135. | Included | Not applicable |
| Valler-Jones, T. (2014). The impact of peer-led simulation on student nurses. British Journal of Nursing. 23(**6**): 321-326. | Excluded | Study describes FLDs exclusively or compares FLDs to no debriefing |
| Verkuyl., M., et al. (2020). Adding self-debrief to an in-person simulation: a mixed-methods study. Clinical Simulation in Nursing. 47: 32-29. | Excluded | Debriefings only including one learner/participant |
| Verkuyl., M., et al. (2019). Comparison of self-debriefing alone or in combination with group debrief. Clinical Simulation in Nursing. 37: 32-39. | Excluded | Multiple exclusion criteria:   - Debriefings only including one learner/participant - Virtual/online/tele-simulation and debriefing study |
| Verkuyl., M., et al. (2018). Virtual gaming simulation: exploring self-debriefing, virtual debriefing, and in-person debriefing. Clinical Simulation in Nursing. 20: 7-14. | Excluded | Multiple exclusion criteria:   - Debriefings only including one learner/participant - Virtual/online/tele-simulation and debriefing study |
| Verkuyl., M., et al. (2020). Exploring debriefing combinations after a virtual simulation. Clinical Simulation in Nursing. 40: 36-42. | Excluded | Multiple exclusion criteria:   - Debriefings only including one learner/participant - Virtual/online/tele-simulation and debriefing study |
| Verkuyl., M., et al. (2021). Using self-debrief after a virtual simulation: the process. Clinical Simulation in Nursing. 57: 48-52. | Excluded | Multiple exclusion criteria:   - Debriefings only including one learner/participant - Virtual/online/tele-simulation and debriefing study |
| Verkuyl., M., et al. (2020). Exploring self-debriefing plus group-debriefing: a focus group study. Clinical Simulation in Nursing. 43: 3-9. | Excluded | Multiple exclusion criteria:   - Debriefings only including one learner/participant - Virtual/online/tele-simulation and debriefing study |
| Verkuyl., M., et al. (2020). Combining self-debriefing and group debriefing in simulation. Clinical Simulation in Nursing. 39: 41-44. | Excluded | Non-empirical study |
| Wilbanks., B. A., et al. (2020). Comparison of video-facilitated reflective practice and faculty-led debriefings. Clinical Simulation in Nursing. 42: 1-7. | Excluded | Debriefings only including one learner/participant |
| 고상진 and 최은희. (2017). Effect of team debriefing in simulation-based cardiac arrest emergency nursing education. Korean Journal of Adult Nursing. 29(**6**): 667-676. | Excluded | Study reported in a language other than English |
| **Studies Identified for Full-text Screening via Other Methods** | **Included/Excluded** | **Reasons For Exclusion** |
| Gantt., L. T., et al. (2018). Comparison of debriefing methods and learning outcomes in human patient simulation. Clinical Simulation in Nursing. 17: 7-13. | Excluded | Debriefings only including one learner/participant |
| Makkink., A. W., & Dreyer, D. J. (2021). Simulation debriefing: a perspective from emergency medical care students at three South African higher education institutions. The Pan African Medical Journal. 38: 97. | Excluded | Non-immersive SLE |
| Miller., E. T., et al. (2018). Asynchronous online debriefing with health care workers: lessons learned. Clinical Simulation in Nursing. 20: 38-45. | Excluded | Virtual/online/tele-simulation and debriefing study |
| Quick., K. K. (2016). The role of self- and peer-assessment in dental students’ reflective practice using standardized patient encounters. Journal of Dental Education. 80(**8**). 924-929. | Included | Not applicable |
| Schreiber, J., et al. (2020). High fidelity simulation with peer debriefing: influence of student observation and participant roles on student perception of confidence with learning and feedback. Journal of Occupational Therapy Education. 4(**2**). | Included | Not applicable |
| Verkuyl., M., et al. (2018). Comparison of debriefing methods after a virtual simulation: an experiment. Clinical Simulation in Nursing. 19: 1-7 | Excluded | Virtual/online/tele-simulation and debriefing study |
| Welke., T. M., et al. (2009). Personalized oral debriefing versus standardized multimedia instruction after patient crisis simulation. Anesthesia & Analgesia. 109(**1**): 183-189. | Excluded | Debriefings only including one learner/participant |
