# Supplementary material for: Exploring in-person self-led debriefings for groups of learners in simulation-based education: an integrative review
Source: Adv Simul (Lond). 2024 Jan 16;9:5. doi: 10.1186/s41077-023-00274-z (PMC10790376; doi:10.1186/s41077-023-00274-z)
Supplement: Supplementary file 3 — Additional file 3. Tabulating themes developed via reflexive thematic analysis. [file 41077_2023_274_MOESM3_ESM.docx]

### Additional File 3: Tabulating themes

| **Sub-themes**  **Studies** | **Role allocation within SLDs** | **Training participants to lead/facilitate SLDs** | **Structure of SLDs** | **Video playback** | **Content of SLDs** | **Combined SLDS + FILDs** | **Previous SBE and clinical experience** | **Professional backgrounds of teams in group SLDs** | **Location and cultural diversity** | **Closing knowledge gaps and reinforcement of erroneous information** | **Resource allocation** |
| --- | --- | --- | --- | --- | --- | --- | --- | --- | --- | --- | --- |
| **Qualitative studies** | | | | | | | | | | | |
| Boet et al. (2016) |  |  | * | * | * |  | * | * |  |  | * |
| **Quantitative RCTs** | | | | | | | | | | | |
| Andrews et al. (2019) | * | * | * | * | * |  |  |  |  | * | * |
| Ha (2020) |  |  | * |  | * |  |  | * | * | * | * |
| Ha & Lim (2018) |  |  | * |  | * |  |  | * | * | * | * |
| Kim & De Gange (2018) | * |  | * |  |  |  |  |  | * | * | * |
| Kündig et al. (2020) |  |  | * |  | * |  |  |  |  | * |  |
| Oikawa et al. (2016) |  |  | * |  | * |  |  | * |  |  |  |
| Rueda-Medina et al. (2020) |  |  | * | * | * | * | * | * |  |  | * |
| Rueda-Medina et al. (2021) |  |  | * | * | * | * |  |  |  |  | * |
| **Quantitative non-RCTs** | | | | | | | | | | | |
| Kang & Yu (2018) |  |  | * | * |  | * |  |  | * | * | * |
| Lee, Kim, et al. (2020) |  |  | * | * |  |  |  |  |  |  |  |
| Na & Roh (2021) | * |  | * |  | * |  |  |  | * |  |  |
| Paige et al. (2021) | * |  | * |  |  |  |  | * |  |  | * |
| Schreiber et al. (2020) | * |  | * |  |  |  | * |  |  |  | * |
| Tutticci et al. (2017) | * | * | * |  |  | * |  |  |  |  | * |
| **Quantitative descriptive studies** | | | | | | | | | | | |
| Curtis et al. (2016) | * |  | * |  | * |  |  |  |  |  | * |
| **Mixed methods studies** | | | | | | | | | | | |
| Boet et al. (2013) |  |  | * | * | * |  | * | * |  |  | * |
| Quick (2016) |  |  | * | * |  |  |  | * |  |  | * |
