# Supplementary material for: Exploring in-person self-led debriefings for groups of learners in simulation-based education: an integrative review
Source: Adv Simul (Lond). 2024 Jan 16;9:5. doi: 10.1186/s41077-023-00274-z (PMC10790376; doi:10.1186/s41077-023-00274-z)
Supplement: Supplementary file 4 — Additional file 4. Exemplar of coding strategy and theme development. [file 41077_2023_274_MOESM4_ESM.docx]

### Additional File 4: Exemplar of coding strategy and theme development

| **Theme** | **Developed Sub-theme** | **Codes** | **Data** |
| --- | --- | --- | --- |
| Challenges of conducting SLDs | Closing knowledge gaps and reinforcement of erroneous information | Facilitators are unable to guide conversation  Facilitators helpless  Faculty authority  Closing knowledge gaps  Erroneous information  Poor practice being taken into clinical practice | “Self-led briefings without an expert may not only have positive effects, as there is a risk of erroneous information may be discussed and negatively influence subsequent performance” ([57], p. 85).  “Alternatively, other than the content of the feedback, students might have preferred reassurance from a faculty authority” ([48], p., 543). |

Example of application of this approach (reflexive thematic analysis) constructing the theme ‘Challenges of conducting SLDs’, in which the researchers are actively interpreting and analysing patterns of data and their meanings, to aid the construction of new knowledge:

The absence of expert facilitators may present a missed learning opportunity, whereby erroneous information could be discussed, consolidated, and reinforced and thereby negatively affect subsequent performance [42,43,45,57]. Furthermore, knowledge gaps may persist into clinical practice if not managed within SLDs, although one study mitigated for this by allowing learners to ask instructors questions and for performance feedback at the end of the SLE [44]. Kundig et al. [57] state that whilst such risky and damaging influences on debriefing outcomes may occur in SLDs, they may be the exception rather than the rule, and could be offset by the beneficial effects of debriefing. However, consistent, and significant student preferences for FLDs over SLDs may indicate learners seeking expert reassurance and accurate debriefing content, which would not be readily available from peers [48,56]. This may be because learners feel they have more opportunity to obtain feedback and reflect on mistakes in FLDs [43,44].
